# Hydrogels and Cubic Liquid Crystals for Non-Invasive Sampling of Low-Molecular-Weight Biomarkers—An Explorative In Vivo Study

**DOI:** 10.3390/pharmaceutics14020313

**Published:** 2022-01-28

**Authors:** Maxim Morin, Skaidre Jankovskaja, Tautgirdas Ruzgas, Joakim Henricson, Chris D. Anderson, Anders Brinte, Johan Engblom, Sebastian Björklund

**Affiliations:** 1 Department of Biomedical Sciences, Faculty of Health and Society, Malmö University, SE-205 06 Malmö, Sweden; maxim.morin@mau.se (M.M.); skaidre.jankovskaja@mau.se (S.J.); tautgirdas.ruzgas@mau.se (T.R.); johan.engblom@mau.se (J.E.); 2 Biofilms–Research Center for Biointerfaces, Malmö University, SE-205 06 Malmö, Sweden; 3 Division of Clinical Chemistry and Pharmacology, Department of Biomedical and Clinical Sciences, Faculty of Health Sciences, Linköping University, SE-581 83 Linköping, Sweden; joakim.henricson@liu.se; 4 Department of Emergency Medicine, Local Health Care Services in Central Östergötland, SE-581 85 Linköping, Sweden; 5 Division of Cell Biology, Department of Biomedical and Clinical Sciences, Faculty of Health Sciences, Linköping University, SE-581 83 Linköping, Sweden; Chris.anderson@liu.se; 6 ImaGene-iT, Medicon Village, SE-223 63 Lund, Sweden; anders@imagene-it.se

**Keywords:** low-molecular-weight biomarker, tryptophan, kynurenine, tyrosine, phenylalanine, tryptophan-to-kynurenine ratio, skin barrier integrity, stratum corneum, natural moisturizing factor, electrical impedance spectroscopy, transepidermal water loss

## Abstract

The molecular composition of human skin is altered due to diseases, which can be utilized for non-invasive sampling of biomarkers and disease diagnostics. For this to succeed, it is crucial to identify a sampling formulation with high extraction efficiency and reproducibility. Highly hydrated skin is expected to be optimal for increased diffusion of low-molecular-weight biomarkers, enabling efficient extraction as well as enhanced reproducibility as full hydration represents a well-defined endpoint. Here, the aim was to explore water-based formulations with high water activities, ensuring satisfactory skin hydration, for non-invasive sampling of four analytes that may serve as potential biomarkers, namely tryptophan, tyrosine, phenylalanine, and kynurenine. The included formulations consisted of two hydrogels (chitosan and agarose) and two different liquid crystalline cubic phases based on the polar lipid glycerol monooleate, which were all topically applied for 2 h on 35 healthy subjects in vivo. The skin status of all sampling sites was assessed by electrical impedance spectroscopy and transepidermal water loss, enabling explorative correlations between biophysical properties and analyte abundancies. Taken together, all formulations resulted in the successful and reproducible collection of the investigated biomarkers. Still, the cubic phases had an extraction capacity that was approximately two times higher compared to the hydrogels.

## 1. Introduction

Local skin diseases such as psoriasis, eczema, and skin cancer change the molecular composition of skin, which is represented by a wide variety of high- and low-molecular-weight (HMW and LMW) compounds (e.g., proteins, lipids, inflammatory mediators, amino acids and their metabolites, etc.) [1,2]. Besides local skin diseases, skin chemistry is also influenced by systemic diseases, such as cancer, diabetes, and atherosclerosis [3]. Therefore, the highly accessible skin surface can serve as a source of biomarkers for various diseases and may be envisaged as a window to the body’s health. Nowadays, the gold-standard procedure for the diagnosis of skin disorders is based on visual inspection followed by histopathologic examination of removed tissue from biopsy [4]. Being an invasive procedure, skin biopsy is associated with several drawbacks, such as risks of infections, aesthetic/visual alterations of the skin, high costs, and long waiting times for patients [5,6,7,8]. Thus, a non-invasive approach for sampling disease-specific biomarkers from the skin surface represents a valuable alternative for clinical diagnostics of various skin disorders.

It is generally known that permeation across the skin barrier depends on the level of stratum corneum (SC) hydration [9,10,11,12,13]. In particular, highly hydrated skin tissue has been shown to result in elevated skin permeability, both for hydrophilic and hydrophobic LMW molecules [12]. Therefore, it is expected that the extraction efficiency of LMW biomarkers by non-invasive sampling from the skin can be increased by hydration. Further, to enable non-invasive sampling of small biomarkers from the skin in a realistic application, it is crucial to ensure high reproducibility, accuracy, and precision. Additionally, in this case, the hydrated state represents a stable endpoint that is achievable as long as the skin site is exposed to an adequate amount of formulation with high water activity (*a*_w_). In this manner, sink conditions are ensured in terms of hydration. Considering these aspects, it is appropriate to employ sampling formulations with high *a*_w_ values that can provide excessive local skin hydration at the sampling site. Further, the formulation should be biocompatible, non-irritative, and have a good ability to absorb the biomarker as well as release it to facilitate analytical quantification. In this work, four water-based formulations with high *a*_w_ values but different physicochemical properties were investigated in terms of their capacity to collect biomarkers from the skin of healthy subjects.

The studied formulations are classified as either hydrogels or liquid crystalline cubic phases. Hydrogels, formed by water-soluble polymers, are widely applied in various scientific and industrial fields [14,15] and also very suitable formulations for the non-invasive sampling of analytes from skin. For example, agarose hydrogels have been successfully employed for collecting skin metabolites from healthy [16] and psoriatic human subjects [17]. Properties that make these formulations attractive are their high content of water, good biocompatibility, and adhesiveness [15,18,19]. Liquid crystalline cubic phases are formed by polar lipids, which can self-assemble spontaneously into a broad variety of nanostructures [20]. In particular, inverse bicontinuous cubic phases have attracted a lot of scientific interest due to their thermodynamically stable structures comprising a single continuous lipid bilayer, which separates two continuous, non-intersecting water channels [21,22,23]. Bicontinuous cubic phases have been used in various applied areas, such as drug delivery [24,25,26,27,28] and encapsulation and crystallization of membrane proteins [29,30,31]. Here, we extend the application of liquid crystalline cubic phases by investigating their potential for collecting LMW biomarkers from skin. In summary, two hydrogel-based and two liquid crystalline cubic phases were prepared and employed in this work. The hydrogels comprised agarose (AGR) and chitosan (CHI), while one bicontinuous cubic phase consisted of the polar lipid glycerol monooleate (GMO) and the other of a mixture of GMO with an addition of the cationic lipid DOTAP (1,2-dioleoyl-3-trimethyl-ammonium-propane) in proportions of 90:10 (wt.%); this mixture is referred to as GTP.

The main objective of this study was to investigate the capacity of these formulations to collect LMW analytes from skin in vivo. The formulations were applied for 2 h on 35 healthy subjects with the aim to collect tryptophan (Trp), tyrosine (Tyr), phenylalanine (Phe), and kynurenine (Kyn) as selected analytes. The reasoning for focusing on these molecules is, in brief, that Trp, Tyr, and Phe are known constituents of the so-called natural moisturizing factor (NMF) of the SC [32,33]. The NMF is a collection of osmotically active free amino acids, amino acid derivatives, and other small polar molecules and specific salts, which represents the biochemical basis for a healthy hydration status of the SC [32,33,34,35,36]. These naturally occurring analytes may serve as references for the diagnosis of various skin diseases. In particular, it has been shown that the ratio between Trp and Kyn (i.e., Trp/Kyn) significantly decreases in cancerous microenvironments [37] and blood levels [38,39], as compared to healthy conditions. Therefore, Kyn was included here as a first attempt to characterize the collected amounts of this analyte from healthy subjects by the different sampling formulations.

Further, transepidermal water loss (TEWL) and electrical impedance spectroscopy (EIS) measurements were conducted pre- and post-sampling. The purpose of these measurements was to assess potential variations of the biophysical properties of the skin barrier and to evaluate how the sampling formulations influenced the skin barrier. Finally, since some of the used formulations may cause skin irritation, the sampling sites were visually screened for signs of irritation.

Taken together, we show that all of the investigated formulations can be successfully used as sampling formulations for extracting LMW analytes from skin with good reproducibility. Still, the novel sampling approach utilizing liquid crystalline cubic phases represents a very promising formulation. In fact, an extraction capacity that was approximately two times higher was achieved by this kind of formulation, as compared to the hydrogel-based formulations. Therefore, great potential is foreseen by employing liquid crystalline cubic phases as formulations for non-invasive sampling of a wide range of LMW biomarkers from skin. Further, this work supports previous studies showing the benefit of characterizing the skin barrier’s biophysical properties with EIS spectroscopy and TEWL for detailing biological variations between skin sites and how topically applied formulations influence the skin barrier properties.

## 2. Materials and Methods

### 2.1. Materials

Glycerol monooleate (GMO, 357 g/mol, RYLO^TM^ MG 19 Pharma, monoglyceride content > 95% *w/w*) was obtained from Danisco Cultor (Brabrand, Denmark), and 1,2-dioleoyl-3-trimethyl-ammonium-propane (chloride salt) (DOTAP, 698.5 g/mol) was purchased from Avanti Polar Lipids Inc (Alabaster, AL, USA). Lipids were used without further purification. High-molecular-weight chitosan (CHI, 310–375 kDa), agarose (AGR, 120 kDa) LE (low electroendosmosis), L-Tryptophan (Trp), L-kynurenine (Kyn), L-phenylalanine (Phe), [^2^H_2_]-L-Phe, L-tyrosine (Tyr), and NaCl were obtained from Sigma-Aldrich (St. Louis, MO, USA). NaH_2_PO_4_·H_2_O and Formic acid (>99%) were obtained from Merck (Darmstadt, Germany). [^13^C_11_, ^15^N_2_]-L-Trp, [^2^H_4_]-L-Tyr and [^13^C_6_]-L-Kyn were obtained from Alsachim (Illkirch-Graffenstaden, France). Ethanol (100% *v/v*) and methanol of HPLC grade were purchased from VWR International (Fontenay-sous-Bois, France). Finn Chambers (Ø 18 mm) were obtained from Navamedic AB (Gothenburg, Sweden). Surgical tape (Micropore^TM^ 2.5 cm × 9.1 m) was obtained from 3M^TM^ (Saint Paul, MN, USA). Wet tissues soaked in 0.9% NaCl solution (Salvequick, wound cleanser) were obtained from Orkla Care AB (Solna, Sweden). All water used was of Milli-Q grade (18.2 MΩ cm).

### 2.2. Study Protocol

**Figure 1:**
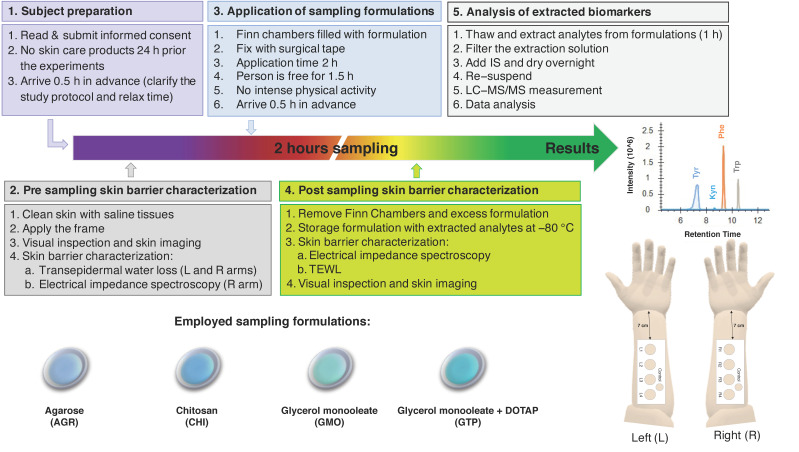
Graphical representation of the study protocol.

Figure 1 shows a graphical representation of the study design. In brief, 35 healthy volunteers (22 females, age range 18–64 years, mean age 35 ± 10 and 13 males, age range 26–61 years, mean age 38 ± 13) with no visible or known dermatological diseases were enrolled in the study after obtaining their informed consent. The study design was approved by the Swedish Ethical Review Authority (Dnr 2020-04943). The study was conducted over three weeks in April 2021 in Malmö, Sweden. There was no drop out during the study.

Test subjects were asked not to use any skin care products 24 h prior to the experiment and not to wear clothes with long sleeves on the day of experiment. Subjects were asked to rest for 30 min before starting the experiments. The skin of the volar forearms was gently cleaned with wet tissues before taping custom-made paper frames between the wrist and cubital fossa on both arms. In this manner, the four sampling sites were fixed on each arm; R1–R4 on the right arm and L1–L4 on the left arm. The paper frames also included one control site for facilitating comparison between treated and untreated sites for any signs of skin irritation by visual inspection.

Prior to the application of sampling formulations, the test sites were visually inspected, and high-resolution images of the test sites were taken. Next, the pre-sampling skin barrier integrity was evaluated by TEWL and EIS measurements. TEWL was measured on both arms, while EIS measurements were only conducted on the right arm in order to investigate if these measurements influenced the quantity of collected analytes. After this, Finn Chambers loaded with the sampling formulations (approximately 300 µL) were applied on the sampling sites and fixed with surgical tape. The order of application between the sampling sites was changed for each test subject by randomization. After 2 h, the Finn Chambers were removed, and the skin surface was gently wiped with a sterile cotton tissue. The reason for employing 2 h as the sampling time was to enable sufficient skin hydration [40], as well as adequate time for extraction of analytes, such as Trp and Kyn [33], while at the same time we were able to avoid an extensively long sampling time for practical reasons. Preliminary in vivo studies performed with CHI, where the sampling time was varied between 0.5 h, 2 h, 4 h, and 8 h, indicated that the amounts increased linearly over time. However, the ratios between the analytes were, overall, relatively stable and not affected by the sampling time (see Figure S1). Thus, 2 h sampling was identified as optimal and used for all sampling formulations. Considering this, all results are presented in units of nmol/cm^2^ instead of as extraction rates (i.e., nmol/cm^2^/h). The formulations were collected in 2 mL plastic Eppendorf tubes, which were then stored in a freezer (−80 °C).

Post-sampling EIS measurements were conducted 5 min after removal of the sampling formulations, while TEWL measurements were performed 8 min after removal. Finally, high-resolution images of the test sites were taken. For more details on the study protocol, see Figure 1.

### 2.3. Preparation of Sampling Formulations and Water Activity (a_w_) Measurements

The CHI hydrogel was prepared by dissolving 200 mg of chitosan in an aqueous solution containing 1% (*v/v*) acetic acid (pH 5.0) and stirred vigorously overnight. The AGR hydrogel (AGR) was prepared by mixing 200 mg of agarose with 9.8 mL of Milli-Q water and heating it up for 2 min in a microwave, while swirling periodically, until the agarose was completely dissolved. Then, 10 mL of hot agarose solution was poured into a Petri dish (Ø 90 mm) and left to cool down at room temperature for 10–15 min with the lid open. Once the agarose gel solidified, the lid of the Petri dish was closed and sealed with tape. The AGR hydrogel was stored at +4 °C until further use (no longer than 16 h). On the day of the experiment, the AGR hydrogel was punched out in circular pieces (ca. Ø 18 mm).

The lipid-based liquid crystalline cubic phases were prepared from water mixed with either GMO or GMO and the cationic lipid DOTAP (this mixture is referred to as GTP). GMO was prepared by melting the lipid at 45 °C using a water bath. Next, water was added to the melted GMO to obtain a water concentration of 45 wt.% and a total mass of approximately 300 mg. The final mixture was kept at room temperature until a clear, non-birefringent, highly viscous bicontinuous cubic diamond (D, Pn3m) phase was formed in the presence of a small amount of excess water (above 40 wt.% water, the Pn3m cubic phase coexists with excess water). GTP was prepared by including DOTAP in the GMO system to obtain a positively charged cubic phase with considerably higher water content (60 wt.%), as compared to only GMO in water (about 40 wt.% water). For this, GMO, DOTAP, and water were mixed with proportions of 36:4:60 wt.%. In brief, DOTAP was first dissolved in 5 mL of ethanol and transferred to GMO. The desired volume of the lipid mixture was transferred to a 2 mL glass vial. Ethanol was removed by an evaporation system (EZ-2 Plus Evaporating System, Genevac Ltd., Ipswich, England, UK) at 35 °C, after which the sample was further dried under vacuum. Next, the required amount of water was added to obtain a final mass of approximately 300 mg. Finally, the lipid–water mixture was left to equilibrate at room temperature for at least a week until a clear, non-birefringent, primitive cubic phase (P, Im3m) was formed. The formation of both cubic phases was confirmed by small-angle X-ray diffraction (SAXD) measurements (see Figure S2).

The water activity (*a*_w_) values for all formulations were determined at 25 °C using the bench-top water activity meter LabTouch-*a*_w_ from Novasina (Lachen, Switzerland). The compositions, *a*_w_ values, physical appearances, and extraction solutions for each sampling formulation used prior to analytical quantification are summarized in Table 1.

### 2.4. Electrical Impedance Spectroscopy (EIS)

In this study, the skin impedance was measured with Nevisense^®^ (Nevisense 3.0, SciBase AB, Stockholm, Sweden) on right arms only (sampling sites R1-R4), pre- and post-sampling. The measurements were performed by pressing the spring-loaded impedance probe, equipped with a non-invasive, five-bar measuring electrode (active area 5 × 5 mm^2^), against the skin site for approximately 8 s. Prior to EIS measurements, the electrode surface was conditioned by wetting it with a saline tissue (0.9% NaCl). The impedance was measured at 35 logarithmically distributed frequencies, ranging between 1 kHz and 2.5 MHz, at four depth settings with a total of 10 permutations. The mean value of the 10 data sets collected for each impedance measurement was used consistently for all data. The applied voltage and resulting current were limited to 150 mV and 75 μA, respectively, ensuring that the test subjects did not experience any uncomfortable sensation [41]. The absolute impedance |Z| at 1 kHz (i.e., |*Z*|_1kHz_) was defined as the skin membrane electrical resistance, simply referred to as skin resistance from here onwards. In addition, the inverse of |*Z*| at 315 kHz (i.e., 1/|*Z*|_315kHz_) was defined as the skin conductance, which is related to skin hydration, similarly to corneometry [40]. A graphical representation of representative impedance data obtained pre- and post-sampling is shown in Figure S3.

### 2.5. Transepidermal Water Loss Measurement (TEWL)

The TEWL was measured by a Delfin VapoMeter^®^ (SWL 5658, Delfin Technologies, Kuopio, Finland). The humidity sensor monitors the change in the relative humidity (RH) inside the chamber and automatically calculates the rate of water evaporation reported in g/m^2^/h. A single TEWL measurement takes less than 30 s, after which the chamber needs to be ventilated before conducting the next measurement. The measurements were performed on both arms (sites R1-R4 and L1-L4) pre- and post-sampling. During the period of study, the humidity and temperature in the test room varied between 30.6 ± 6.3% RH and 20.9 ± 1.1 °C, respectively.

### 2.6. High-Resolution Imaging

High-resolution images of the sampling sites were obtained pre- and post-sampling in order to evaluate appearance of skin irritation. Images were obtained with Nikon D300 (Nikon Corporation, Tokyo, Japan) using a focal length 90 mm and a F-number of f/8.

### 2.7. Extraction of Analytes from the Sampling Formulations

First, the samples were taken out of the freezer (−80 °C) and thawed at room temperature before adding 1 mL of the extraction solution specified in Table 1. Considering that the sampling formulations have different physicochemical properties, minor modifications of the extraction solutions were used with the aim to separate/precipitate the sampling material from the hydrophilic extraction media containing the solubilized analytes in stable forms [33]. Next, all samples were shaken at 400 rpm for 1 h, followed by centrifugation (12,000× *g* for 15 min at 20 °C). After that, the supernatants from the samples containing AGR, GMO, and GTP were filtered with a syringe filter (13 mm, w/0.2 µm PTFE membrane, VWE International). For the CHI hydrogel samples, which were only partly precipitated, syringe-based filtration was not possible. Instead, these samples were centrifuged (12,000× *g* for at least 30 min at 20 °C) with centrifugal filters (10 kDa cut-off, PES modified, VWR International). Next, isotopically labelled analytes (5 µM in 20 μL aliquots) were added to 380 µL of the filtered extraction solution. Subsequently, the solution was concentrated 10 times by evaporation using the Genevac system, after which 40 µL of H_2_O:MeOH (80:20% (*v*/*v*)) was added. From this solution, 30 μL was injected into the LC-MS/MS system for quantification.

### 2.8. Liquid Chromatography Mass Spectrometry Analysis (LC-MS/MS)

The quantification of Trp, Tyr, Phe, and Kyn was achieved by a mass spectrometer system (Waters QAA009 Micromass Quattro microTandem Quadrupole) equipped with an electrospray ionization (ESI) source and connected to an HPLC System (Waters 2795 Alliance High Throughput). The analytes were separated on a Kromasil C18 column from ES Industries (West Berlin, Germany) with 100 Å pore size, 5 μm particle size, and dimensions of 4.6 mm (i.d.) × 250 mm (l). Solvents A (0.1% formic acid in water) and B (0.1% formic acid in methanol) were used to create a gradient to elute the analytes. Separation was performed in a 25 min linear gradient. The gradient profile was as follows: 10% of solvent B was increased to 90% over 15 min at flow rate of 0.5 mL/min and held at 90% B for 5 min at the flow rate of 0.8 mL/min; then solvent B was decreased to 10% in 0.1 min and kept at 10% B at the flow rate of 0.5 mL/min for 4.9 min. The quantification of analytes was carried out in multiple-reaction monitoring (MRM) analytical mode, while operating at positive polarity. The capillary voltage was set to 3.05 kV. The source temperature was kept at 110 °C. The flow of desolvation gas was set to 900 L/h, cone gas flow to 25 L/h, and the desolvation temperature raised to 400 °C. MRM transitions were acquired in Q1 and Q3, and the collision gas pressure in Q2 was set to 8.8 × 10^−4^ Torr. The interchannel and interscan delays were set to 0.1 s. Dwell time was 0.005 s for all MRM transitions. The span window was set to 1 Da. The MRM transitions and chromatographic parameters are detailed in Table S1. Quantification of the analytes was based on the ratio of the peak areas corresponding to the light and heavy ions. Peak integration was performed automatically by using Skyline v3.5 software (MacCoss Lab Software, Washington, DC, USA). All data were manually checked to confirm that the software correctly assigned the peaks. The precision, accuracy, limit of detection (LOD), and limit of quantification (LOQ) of the method are summarized in Table S2. The concentrations of Tyr, Phe, and Trp from the collected samples were always detected above LOQ. However, the concentration of Kyn was detected below LOQ but above LOD. Further, the analysis of the standard solution with the lowest Kyn concentration (i.e., 0.25 µM), showed poor precision (i.e., coefficient of variation of 44%) and accuracy (i.e., recovery of analyte vs. internal standard of 84 ± 37%), see Table S2. In other words, all results of Kyn should be treated with caution as they represent estimated values.

### 2.9. Statistical Analysis

All statistical analyses were performed with RStudio v 1.3.1093 (Boston, MA, US) [42]. Data are presented as box plots in combinations with strip charts and reported as mean values ± standard deviation (SD), calculated based on the number of observations (*n*) after outliers had been removed. The boundaries of the boxplot represent the first (Q1) and third (Q3) quartiles, the thick bar shows the median value, and the vertical line reports the range of the values observed. The test for outliers was performed using the interquartile range (IQR) method, in which values 1.5 × IQR lower/higher than Q1/Q3 were considered as outliers.

The distribution of the data was tested for normality by performing Shapiro–Wilk and Kolmogorov–Smirnov tests as well as by inspecting quantile–quantile (QQ) plots. Homogeneity of the variances was checked by performing Levene’s test. A two-tailed Student’s *t*-test was used to compare differences between two unrelated groups. When neither homogeneity of variances nor assumption of normality were fulfilled, a non-parametric Wilcoxon rank sum test was applied. The difference in the amounts and the ratios of analytes collected from the left and right arms were not significantly different. Therefore, in order to overcome intraindividual variability, further statistical analysis tests were carried out on the average values between the left and right arms. One-way ANOVA with multiple comparisons (Tukey’s test) was used to analyze differences between sampling formulations and sampling sites. Alternatively, if assumptions of homogeneity of variances were not fulfilled, Kruskal–Wallis followed by Wilcoxon rank sum test with false discovery rate correction (FDR) were performed. ANCOVA analysis was used to evaluate the effect of age as covariable when testing differences between males and females. Linear regression analysis was performed for the relationship between the quantity of analytes. Further, correlations between |Z| at 1 kHz, skin conductance, TEWL, and absolute quantities of sampled analytes were evaluated by Spearman’s rank test. The significance levels used were: * *p* < 0.05, ** *p* < 0.01, *** *p* < 0.001.

## 3. Results

The overall aim of this work was to investigate the capacity of different sampling formulations (see Table 1) to collect Trp, Tyr, Phe, and Kyn from skin. To allow for an unbiased comparison of the sampling formulations, a prerequisite was that the skin sampling sites had to have a relatively similar overall skin status. Obviously, a certain degree of biological variation was expected considering that the group of test subjects included 22 females and 13 males with an age span between 18 and 64 years. One approach to evaluate the skin status is to determine the biophysical properties of the sampling sites, which may influence the amounts of extracted biomarkers. Further, it is important to ensure that any differences related to biological variations are minimized by employing a random distribution of the formulations between the sampling sites. To assess these kinds of issues, firstly, the biophysical properties of the skin sites were compared between females and males and between sampling sites, irrespective of sex. This comparison was made both before and after application of the sampling formulations. Secondly, a similar evaluation was performed to assess the biological variability of the collected amounts between females and males and between sampling sites, irrespective of the sampling formulation.

### 3.1. Characterization of the Skin Barrier Biophysical Properties by EIS and TEWL

The skin barrier biophysical properties were assessed with EIS and TEWL pre- and post-sampling. The results from these measurements are presented in detail in Table S3, while a summary follows below.

### 3.2. Skin Barrier Biophysical Properties Pre- and Post-Application of Sampling Formulations

As a first comparison, the pre-sampling skin resistance (i.e., |*Z*|_1kHz_), skin conductance (i.e., 1/|*Z*|_315kHz_), and TEWL values, for females and males, including an ANCOVA analysis taking into account age as covariable, are shown in Figure S4. The skin resistance for females was significantly higher as compared to males. Further, females had somewhat lower conductance values, but this difference was not significant as compared to males. In contrast to the EIS measurements, which were only conducted on the right arms, the TEWL was assessed on both arms of the test subjects. For both arms, females had significantly lower TEWL, as compared to males. However, no significant TEWL differences were observed between the left and right arms within the group of females nor the group of males. Importantly, the outcome of the ANCOVA analysis shows that the effect of age cannot be excluded, implying that the observed differences may be due to a combination of age and sex variations.

After application of the sampling formulations, the skin resistance decreased, while both the skin conductance and the post-TEWL increased, as compared to the initial values for females and males (Figure S4). Further, no statistical differences between females and males were identified. Here, it is important to point out that the post-TEWL values mainly reflect the skin surface water loss, which is related to evaporation of water superficially absorbed by the skin barrier from the applied formulation.

To explore the variability of the biophysical properties of the skin barrier between different sampling sites, a decision was made to analyze the results obtained from each sampling site, irrespective of sex (Figure S5). In general, no significant differences were observed, neither for the skin conductance nor the TEWL values between the different sites. However, the skin resistance values measured at sampling site R1 were significantly lower as compared to the values measured at the sites R3 (*p* = 0.001) and R4 (*p* = 0.003). Further, the results in Figure S5 confirm that application of the sampling formulations for 2 h results decreased skin resistance, while both the skin conductance and the post-TEWL increased, as compared to the initial values for each sampling site.

In addition, these data were analyzed by means of the difference between the pre- and post-values with the aim of omitting interindividual variability, see Figure S5. Overall, the results from this analysis confirm that the sampling sites were affected in a similar manner.

### 3.3. The Effect of Sampling Formulations on the Skin Barrier Biophysical Properties

**Figure 2:**
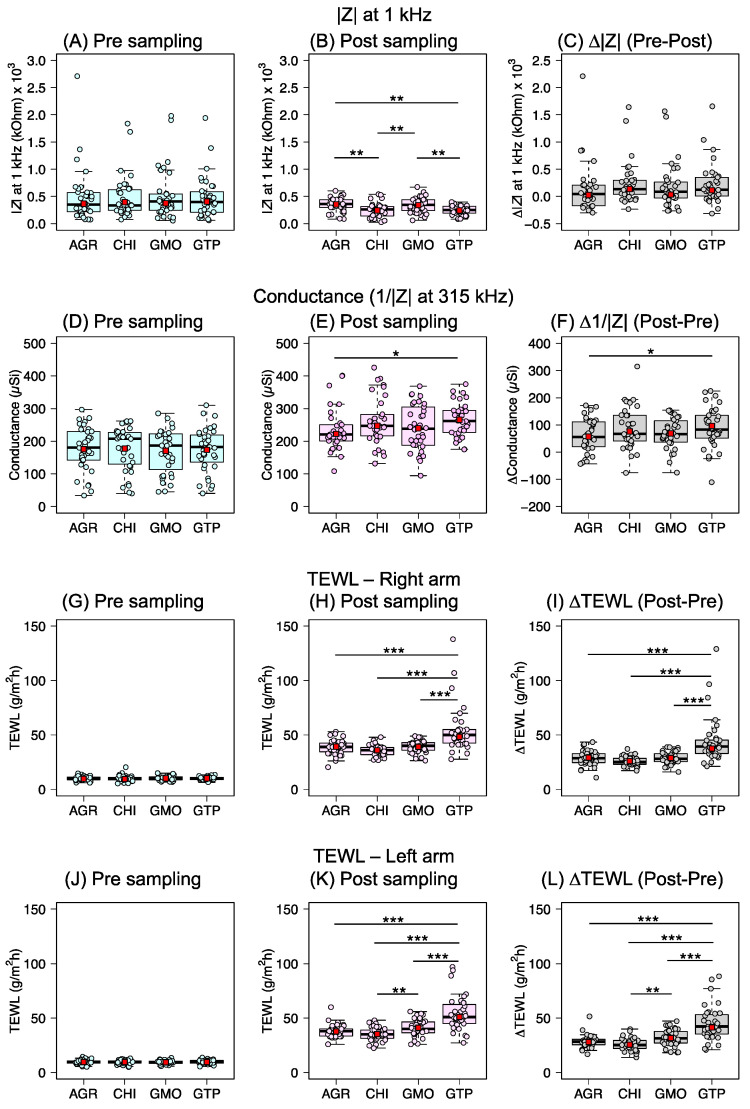
Comparison between pre- (left panel, (**A**,**D**,**G**,**J**)) and post- (middle panel, (**B**,**E**,**H**,**K**)) skin barrier biophysical data obtained at skin sites grouped according to sampling formulations, including the difference between corresponding pre- and post-data (right panel, (**C**,**F**,**I**,**L**)). Skin resistance values represent |Z| at 1 kHz, and conductance values represent 1/|Z| at 315 kHz. The significance levels used were: * *p* < 0.05, ** *p* < 0.01, *** *p* < 0.001. See Table S4 for a compilation of *p*-values derived from a statistical analysis of the data presented in this figure (one-way ANOVA with multiple testing, Tukey test).

The results from the initial measurements in Figure 2A,D,G,J illustrate that the observed differences of the skin barrier’s biophysical properties between females and males and between skin sampling sites, observed pre-sampling, are cancelled out by the randomization protocol. In other words, there were no statistical differences in the skin resistance, conductance, and TEWL between the sampling sites. This is an important observation implying that the different sampling formulations were applied, on average, on skin sites with similar biophysical properties.

In general, the results in Figure 2B,E (i.e., post-sampling EIS data) show that the skin resistance decreased, and the conductance increased, which indicate that application of the sampling formulations, which all have high water activities (see Table 1), leads to elevated skin hydration. In particular, this effect was noticed in the post-skin resistance data after application of CHI and GTP (Figure 2B) and in the post-conductance data for GTP (Figure 2E). However, the difference between the pre- and post-resistance values (Figure 2C), which act to minimize the interindividual variability, did not show any significant effects. Similarly, the statistical difference between AGR and GTP in the post-conductance data (Figure 2E, *p* = 0.015) almost disappeared when comparing the post- and pre- conductance data (Figure 2F, *p* = 0.048). In general, this implies that all formulations affected the skin resistance and conductance similarly (i.e., overall similar hydration effects). As expected, the post-TEWL (i.e., measured after application of the sampling formulations) was significantly higher as compared to the initial TEWL values, irrespective of formulation. This can be explained by evaporation of residual water leftover from the sampling formulations. In particular, this effect is clearly observed in the data comparing the difference between the post- and pre-values (Figure 2I,L), which in principle should only reflect skin surface water loss.

### 3.4. Comparison of Analyte Amounts Collected from Females and Males

Speculatively, the analyte abundance in skin may be subject to a biological variability between females and males. Therefore, a comparison addressing this issue was performed (*t*-test and ANCOVA, see Figure S6). As illustrated, based on the *t*-tests, no significant differences for Tyr, Phe, and Kyn were observed. However, a significantly higher amount of Trp was collected from the females (0.82 ± 0.39 nmol/cm^2^, *n* = 88), as compared to the males (0.63 ± 0.34 nmol/cm^2^, *n* = 49), see Figure S6E. Due to this, the Tyr/Trp and Phe/Trp ratios were significantly lower in the samples collected from females, as compared to the males (see Figure S6I,K, respectively). Moreover, a minor, but significant, difference was observed when comparing the Phe/Tyr ratio between females and males (Figure S6M). However, the ANCOVA analysis (Figure S6), taking age into account as a covariable, showed a strong reduction in the observed differences, indicating that variations in both age and sex influence the analyte abundancies in general.

### 3.5. Comparison of Analyte Amounts Collected from Left and Right Arms

The application of the different sampling formulations, between the four sampling sites of each arm, was randomized for each individual subject. However, the order of application of the sampling formulations was identical for both arms. This design was utilized in order to enable a general assessment of the variability by comparing the collected amounts from the left and right arms (Figure S7A–D). Here, it may be noted that this comparison is independent of formulation type and combines data from both females and males. In brief, this comparison show that the collected amounts of all analytes were similar for both arms, which means that the sampling approach, extraction procedure, and quantification analysis are highly reproducible. Further, no statistically significant differences were observed between the different ratios of analytes collected from different arms (Figure S7E–H).

Even though the analyte abundancies in the skin sites from different arms were concluded to be similar, the collected amounts were slightly higher when extracted from left arms. For example, the average amount of Tyr collected from the left arm was 2.92 ± 1.70 nmol/cm^2^ (*n* = 137), while the corresponding amount from the right arm was 2.88 ± 1.76 nmol/cm^2^ (*n* = 140). A reasonable explanation for this is that EIS measurements were only performed on the right arms, which may have led to a minor removal of analytes from these skin sites during the measurement when the prewetted electrode was in contact with the skin surface.

### 3.6. Comparison of Analyte Amounts Collected from Different Sampling Sites

Even though the application of the different sampling formulations was randomized for each individual subject, the application incidences of the formulations were not equally distributed (see Table S5). Therefore, prior to comparing the efficacy of the formulations to collect biomarkers, the biological variability between the sites was examined by focusing on each formulation separately (see Figure S8). This comparison shows that there were no statistically significant differences between the sites when the analytes were collected by AGR, CHI, and GTP. However, in one case (i.e., GMO), statistically significant differences were observed between the amount of Tyr and Phe from three different sampling sites (Figure S8I,J, respectively). Here, it should be noted that the application frequency of GMO was relatively uneven on these particular sites (see Table S5). For example, GMO was applied 13 times on the skin sites L1/R1 and only 6 times on the sites L2/R2. Thus, it is likely that the observed differences were due to varying analyte abundancies between test subjects, rather than differences between sampling sites. In support of this, a control experiment was performed (with AGR) where the amounts of the analytes from the same individual but different sites had a coefficient of variation (CV) below 20%. This was compared to the CV values for the quantities of analytes collected from the same site but different individuals, which resulted in CV values above 40%. Taken together, it is reasonable to conclude that the analyte abundancies for all sampling sites of a particular test subject are similar, which allows for an unbiased comparison of the extraction capacities of the sampling formulations.

### 3.7. Assessment of the Capacities of the Sampling Formulations to Collect Analytes

**Figure 3:**
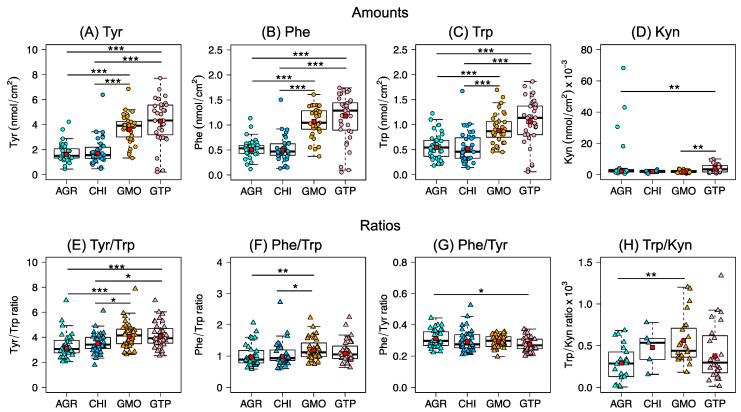
Comparison of the capacity of AGR, CHI, GMO, and GTP to collect Tyr (**A**), Phe (**B**), Trp (**C**), and Kyn (**D**) from the skin surface of healthy volunteers. Ratios between collected amounts of Tyr/Trp (**E**), Phe/Trp (**F**), Phe/Tyr (**G**), and Trp/Kyn (**H**). The significance levels used were: * *p* < 0.05, ** *p* < 0.01, *** *p* < 0.001. See Table S7 for a compilation of *p*-values derived from a statistical analysis of the data presented in this figure (one-way ANOVA with multiple testing, Tukey test).

The different sampling formulations were assessed by performing statistical analysis on the quantities of analytes collected from both arms of 35 healthy volunteers, where each sampling formulation was applied on 70 different sampling sites. Due to the fact that the amounts collected from either left or right arms were identical (see Figure S7), the statistical analysis was performed based on average values (i.e., *n* = 35). The comparison between the different sampling formulations is shown in Figure 3, where outliers (data points outside the minima/maxima of the boxplots) were taken away from the dataset for the statistical analysis. This was carried out in order to minimize the influence of biological variability and focus on the capacity of different sampling formulations for collecting the selected analytes. For clarity, Table S6 includes a summary of all data (amounts in Table S6A and ratios in Table S6B), with and without outliers. Importantly, irrespective of inclusion or exclusion of outliers, the conclusions are the same.

The results in Figure 3 clearly show that the capacity of the lipid-based cubic phases (i.e., GMO and GTP) to collect Tyr, Phe, and Trp was significantly higher as compared to the AGR and CHI hydrogels. Further, a comparison of the collection capacity between the cubic phases shows that GTP collected higher amounts of Tyr, Trp, and Phe, as compared to GMO. Additionally, for the specific case of Kyn, significantly higher quantities were collected by GTP, as compared to GMO and AGR. However, it should be underlined that all Kyn results are estimations since the concentrations of this analyte could not be quantified with adequate accuracy and precision (i.e., [Kyn] < LOQ). Nevertheless, the incidence to collect Kyn was similar for the lipid-based formulations and the AGR hydrogel, while the incidence was lower for the CHI hydrogel. In brief, Kyn was detected in 54% of the total amount of samples collected by GMO, while the corresponding percentage was 49% in the case of AGR, 41% in the case of GTP, and only 10% for CHI. A comparison between the hydrogels showed no statistically significant differences between the collected quantities of the analytes. Taken together, the lipid-based formulations collected approximately two times more Tyr, Phe and Trp, as compared to the hydrogels (see Table S8 for a detailed summary).

In addition, a comparison between different analyte ratios corresponding to the different sampling formulations is shown in Figure 3E–H. Importantly, by analyzing the analyte ratio, instead of their absolute quantities, the overall variability between the sampling formulations decreased considerably (see Table S9 for a detailed summary of variation coefficients). However, some significant differences were observed in the data set of analyte ratios (see Figure 3E–H), which are related to minor variations of the collected amounts of different analytes by the sampling formulations.

**Figure 4:**
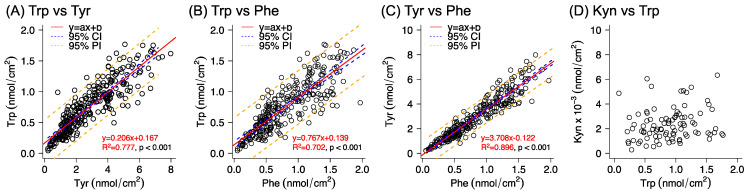
Correlation plots (**A**–**D**) between the collected amounts of analytes from individual skin sampling sites. The plots include regression line, the 95% confidence interval (95% CI), and the prediction interval (95% PI). In case of Kyn vs. Trp (**D**), the R^2^ was 0.033 and *p* = 0.080. Number of data points: *n* = 277 for Tyr, *n* = 274 for Phe, *n* = 277 for Trp, and *n* = 93 for Kyn.

As a complement to the evaluation of analyte ratios, correlation plots of analyte abundancies are presented in Figure 4. The results show strong correlations between the quantities of Tyr, Phe, and Trp with linear regression coefficients (R^2^) above 0.7 in all cases. However, no correlation was found between the collection of Trp and Kyn, which also gives rise to higher variation of the Trp/Kyn ratio (see Table S9). This is most likely due to the overall very low amounts of Kyn detected in the samples collected from healthy subjects in combination with the inadequate analytical quantification (i.e., [Kyn] < LOQ).

### 3.8. Correlation between Skin Barrier Biophysical Properties

The skin resistance, conductance, and TEWL reflect the biophysical properties of the skin barrier and provide useful information on how the skin barrier is affected by the sampling formulation. In order to analyze correlations between these parameters, Spearman’s rank correlations were performed for all data collected pre- and post-sampling (see Figure S9). In brief, strong negative correlations were observed between the skin resistance and conductance, both when comparing pre- as well as post-sampling measurements. This finding concludes that the electrical resistance of the skin barrier is strongly correlated to the hydration degree (as determined by the conductance). Further, weak correlations between the initial skin resistance and TEWL, as well as the initial skin conductance and TEWL, were observed. Even though these correlations were weak, they show that a high electrical resistance and a low conductance of the skin barrier are associated with low TEWL values. This confirms that all these parameters can be used to evaluate the integrity of the skin barrier in some capacity. As expected, no correlations were observed when comparing the skin resistance or the skin conductance with the post-sampling TEWL, which can be explained by the domination of the latter parameter by the evaporation of residual water from the sampling formulations, which does not reflect the intrinsic skin barrier properties.

### 3.9. Correlation between Skin Barrier Biophysical Properties and Analyte Abundancies

To evaluate existing correlations between the various skin barrier biophysical parameters and the collected amounts of analytes, an extensive and explorative correlation analysis was performed (see Figure S10). This analysis included the assessment of correlations by combining data from all sampling formulations (Figure S10A), as well as dividing the data for each individual sampling formulation separately (Figure S10B–E). Since Tyr, Phe, and Trp are constituents of the NMF pool [32,36] and also shown in Figure 4 to strongly correlate in terms of collected amounts from individual sampling sites, it was decided to sum up the collected amounts of these analytes and introduce a more general representation of these data, which we simply refer to as NMF (see Figure S10A–E).

**Figure 5:**
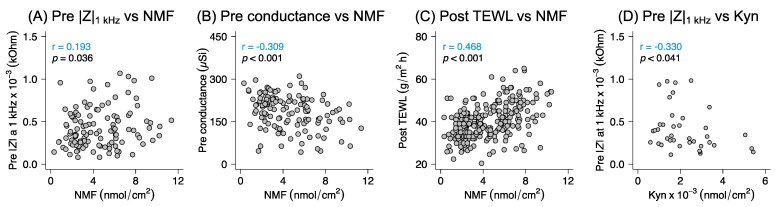
Spearman’s correlation plots between the biophysical properties of the skin barrier and the collected amounts of analytes (i.e., NMF). Correlation between the initial skin resistance (**A**), the initial skin conductance (**B**), post- TEWL (**C**), and the total quantity of collected NMF. Correlation between the collected quantity of Kyn and the initial skin resistance (**D**).

Starting with the correlations based on the data from all sampling formulations combined, a weak positive correlation was found between the NMF quantities and the initial skin resistance values (Figure 5A), while a weak negative correlation was observed between the NMF quantities and the initial conductance measurements (Figure 5B). Moreover, a weak positive correlation was found between the collected NMF quantities and the post-sampling TEWL (Figure 5C). No other significant correlations were observed between the NMF quantities and the skin barrier measurements, neither pre- nor post-sampling (Figure S10A). Interestingly, in the case of Kyn, a weak negative correlation was observed between the initial skin resistance values and the collected amounts of this molecule from all formulations combined (Figure 5D). However, considering that the collected amounts of Kyn were detected below the LOQ, this result must be confirmed by future studies with more accurate and precise analytical methods for Kyn quantification.

With respect to the correlation analyses for each individual sampling formulation (Figure S10B–E), the correlations were in general improved for CHI and GTP (Figure S10C,E), as compared to the correlations based on data from all formulations combined. On the other hand, for AGR or GMO, the corresponding correlations were inferior (Figure S8B,D). Interestingly, these observations imply that the data can be grouped according to the charge status of the formulation, where CHI and GTP represent positively charged formulations with improved correlations (Figure S10F) and AGR and GMO represent non-charged formulations with mediocre correlations (Figure S10G). However, the reason for these correlation patterns is not straightforward.

## 4. Discussion

### 4.1. The Importance of Sufficient Skin Hydration for Efficient Collection of Biomarkers

The extraction characteristics of the skin membrane depend on the level of skin hydration [9,10,11,12,13], as a highly hydrated skin barrier is known to have elevated permeability for both hydrophilic and hydrophobic substances [12]. Further, the degree of skin hydration can be regulated externally by application of formulations with high water activity [12,13,43]. Therefore, in order to ensure optimal hydration of skin barrier, all sampling formulations were designed with high water activities to enable sufficient skin hydration (Table 1). Further, the study protocol (Figure 1) included initial measurements of the skin barrier biophysical properties by EIS and TEWL to ensure that all sampling sites were similar in terms of skin conductance, resistance, and TEWL (see Figure 2). Importantly, these measurements were also performed post-sampling to ensure that the sampling sites were sufficiently hydrated. In conclusion, all formulations resulted in significantly increased skin conductance values and decreased skin resistance values (Figure 2). In other words, these results imply that the skin barrier was sufficiently hydrated during the 2 h long application period of the sampling formulations, which was desired in order to reach stable and efficient extraction conditions for the investigated analytes from skin. These results are in the good agreement with our previous study, showing that 1 h of skin hydration with an aqueous solution (*a*_w_ ≈ 0.995) is sufficient to stabilize the electrical properties of the skin barrier in vivo of healthy subjects [40].

### 4.2. Prerequisites for an Unbiased Comparison of the Different Sampling Formulations

Caution was taken to ensure that the applied formulations were appropriately distributed on the skin sampling sites to enable an unbiased comparison of the capacity of the formulations to collect the analytes. Several assessments were performed to evaluate the biological variations, e.g., between females and males, between left and right arms, and between the skin sites of individual test subjects. From these assessments, some interesting findings were observed. For example, the comparison between females and males shows that the initial measurements of skin resistance, conductance, and TEWL were observed to be different (Figure S4). In line with these findings, Nicander et al. reported significant differences between males (*n* = 62) and females (*n* = 69) in electrical impedance indices measured on the forearms of healthy volunteers [44]. Moreover, they found higher TEWL values on the forearms of males, as compared to females [44]. There are, however, several contradictions in the literature on this topic [45] and, in general, most studies could not establish differences for TEWL values between males and females [46,47,48]. In the case of this study, it is possible that the observed discrepancies may be related to the fact that the test subjects included more females (*n* = 22), which were also slightly younger (average age = 35 ± 10), as compared to the males (*n* = 13, average age = 38 ± 13). This reasoning is supported by the ANCOVA analyses, indicating that the observed differences may be due to a combination of age and sex variations. Importantly, the impact of the observed differences in analyte abundancies were cancelled out by the randomization protocol. This conclusion means that any observed differences between the sampling formulations are strictly related to the properties of the formulations and not to biological differences between sites and test subjects.

### 4.3. Liquid Crystalline Cubic Phases Are Excellent for Non-Invasive Skin Sampling of Low Molecular Weight Biomarkers

As shown in Figure 3A–D, the capability to collect high amounts of analytes from skin was significantly higher for the liquid crystalline cubic phases (GTP and GMO), as compared to the hydrogels (AGR, CHI). In particular, the application of GTP resulted in the highest quantities of collected analytes, which could be due to several reasons. Firstly, the lipid-based liquid crystalline cubic phases are expected to have a good capability to accumulate and retain the analytes within their interconnected bicontinuous oil and aqueous channels. In particular, this microstructure provides a tremendous surface area per volume where the analytes can preferentially partition according to their lipophilic–hydrophilic characteristics. Considering that the Trp, Tyr, and Phe molecules have relatively non-polar amino acid side groups and a highly polar zwitterionic amino acid center, it is likely that these analytes partition in the interface of the hydrophilic headgroups and the hydrophobic acyl chains of the cubic phases [49]. This property of cubic phases could potentially be extremely useful for sampling a wide range of biomarkers with various physicochemical characteristics. In other words, hydrophilic analytes may preferentially partition in the aqueous water channels, amphiphilic analytes in the headgroup-acyl chain interfacial regions, and hydrophobic analytes inside the oil channels. In contrast, the hydrogels have less well-defined microstructure and lack a similar hydrophilic–hydrophobic interface. In addition, considering that the diameter of the water channels of these cubic structures are relatively large (>4 nm), larger biomarkers could also potentially be collected from the skin surface.

Another aspect that can influence the collected amounts of analytes is the presence (or absence) of ionic charges of the sampling formulation. For example, the highest quantities of analytes were collected by the cubic phase GTP, which was doped with the positively charged DOTAP lipid. Thus, incorporation of DOTAP in the GMO base may facilitate electrostatic interactions between the positively charged amine group of DOTAP and the negatively charged carboxylate group of the analytes, which might explain the enhanced collection observed for GTP, as compared to GMO. Speculatively, it is not unlikely that the charged DOTAP lipid may diffuse from the cubic phase and partition to some extent into the skin barrier where it may induce temporal changes of the lipid matrix of the SC, similar to the mode of action of some penetration enhancers [50]. In support of this, the transdermal permeation of the NSAID meloxicam (351 Da), from liposome formulations with varying surfactant charges (anionic, neutral, and cationic), was previously shown to be significantly enhanced by the incorporation of positively charged species in the liposome system [51]. In line with this reasoning, the skin resistance measured on the skin sites that were exposed to GTP was significantly lower, as compared to the GMO (see Figure 2B). A similar effect on the skin resistance was also observed for CHI (see Figure 2B), which also carries positively charged amine groups. However, in the case of CHI, the charged functional groups are covalently attached to the polymer backbone, which excludes a similar mechanism of action. Therefore, it is more likely that the observed decrease in the skin resistance observed for CHI is related to some other mechanism, such as increased skin hydration, which is known to decrease the skin resistance [40,43]. In fact, in our previous study it was found that the CHI hydrogel loses up to 70% of its initial water content after 2 h of skin application (Jankovskaja et al., 2021, unpublished results), which supports this reasoning. The higher loss of water from the CHI formulation can also explain why this hydrogel proved to have an inferior collection capacity since it shrinks in volume during the 2 h application, thereby limiting the enrichment of the hydrophilic analytes.

Another aspect that can influence the collection capacity is related to the sampling formulation’s ability to fill skin furrows and appendages and ensure a high coverage of the area of the skin sampling site. With respect to this, the liquid crystalline cubic phases, being relatively viscous, are expected to have a high ability to conform to the skin topography, while the AGR hydrogel, being a semisolid formulation, may have a lower ability to adjust to skin topology.

### 4.4. Analyte Ratios Is Preferable for Minimizing Biological and Sampling Procedure Variability

A general observation based on the results in Figure 3E–H is that the ratios were similar for all formulations, which illustrate that the concept of using analyte ratios as a potential biomarker is more reliable as compared to probing individual analytes. In this manner, differences related to sampling protocols and sampling set-ups are minimized. Moreover, the variability of the analyte ratios is significantly lower as compared to the absolute quantities of the collected analytes (Figure 3A–D). A closer analysis of the biological variation of the absolute quantities shows that the inter-individual variation, CV (%), for Tyr, Phe, and Trp was between 23–51%, see Table S9. Notably, the variation for the ratios between these analytes (i.e., Tyr/Trp, Phe/Trp and Phe/Tyr) is significantly reduced to values between 14–33%.

The analyte ratios reported here (e.g., in case of AGR 3.1 for Tyr/Trp, 1.0 for Phe/Trp, and 0.3 for Phe/Tyr, see Table S6B) are in general agreement with our previous studies (Jankovskaja et al., 2021, unpublished results), as well as with the work of others. For example, in samples collected by tape stripping, the reported ratios of Tyr/Trp, Phe/Trp, and Phe/Tyr were approximately 2.5, 1.0, and 0.4, respectively [52]. In case of sampling by reverse iontophoresis, the ratios determined in the samples collected at the cathode were approximately 2.3 for Tyr/Trp, 1.1 for Phe/Trp, and 0.5 for Phe/Tyr [52]. Moreover, sampling by holding 1 mL of PBS in the palm for 2 min resulted in Tyr/Trp, Phe/Trp, and Phe/Tyr ratios of around 4.1, 1.9, and 0.9, respectively [53]. These results show that, despite different sampling strategies and analytical procedures, the obtained ratios are very robust. In summary, the overall low variations of the ratios reported here are promising and show that employment of the analyte ratio as a potential biomarker represents a suitable approach to use in a clinical situation for skin disease diagnostics.

In the special case of the Trp/Kyn ratio, the variation was 51–77%, which is similar to the variation observed for the estimated amounts of Kyn (40–70%). The reason why the variation remained large, going from individual analyte amounts to their corresponding ratios, could in this case be related to the inadequate analytical quantification of Kyn (i.e., [Kyn] < LOQ). On the other hand, the absence of correlation between the collected amounts of Trp and Kyn from healthy skin is in line with the fact that the origin of these analytes differs. In brief, Trp is naturally present in skin as a NMF component, while Kyn is a downstream metabolite of Trp [54]. In particular, a decrease in the Trp/Kyn ratio is a recognized biomarker for cancer [37,38,39]. However, this kind of biomarker, based on a single ratio (e.g., Trp/Kyn), is obviously more sensitive to biological variations, as compared to the case of probing multiple analyte ratios. The strong correlations observed in Figure 4 between the abundancies of Trp and Tyr, Trp and Phe, and Tyr and Phe could thus be used as a quality control to assess the variability of the collected quantity of Trp on healthy human skin and to help to increase the reliability when assessing the Trp/Kyn ratio as a skin cancer biomarker. In conclusion, a more reliable strategy for using the Trp/Kyn ratio as a cancer biomarker is to include several amino acids in order to account for biological variations originating from NMF [36,55]. Further, in addition to NMF compounds, other analytes could be identified by employing untargeted metabolomics, in combination with multivariate analysis, with the aim of finding correlations of all detectable metabolites, including their corresponding ratios. This was, however, beyond the scope of this study.

### 4.5. Correlation between NMF and Biophysical Properties of the Skin Barrier

Formation and maintenance of a healthy skin barrier relies on synthesis of key lipids (e.g., very-long-chain fatty acids, ceramides, cholesterol), proteins (e.g., keratin filaments, corneodesmosomes), and NMF components, as well as the assembly of these molecular components into the optimal brick-and-mortar barrier structure [56]. The lipids are required to form the continuous extracellular lipid matrix (i.e., the mortar), while corneocytes, including the NMF components that are mainly residing inside the corneocytes, represent the main building block of the SC (i.e., the bricks) [32,36]. The fact that a healthy skin barrier should contain a certain level of NMF content [32,36], as well as a certain degree of barrier integrity, which can be assessed with EIS and TEWL [57,58], implies that these parameters should be correlated. Thus, a decision was made to analyze all data obtained in this work in an explorative approach looking for possible correlations (see Figure S10 for a detailed summary of these correlations). Taken together, a high amount of extracted NMF was associated with a low initial conductance. This indicates that a less hydrated, but still healthy, skin barrier contains more NMF. Further, a high skin resistance was related with a high amount of NMF, which implies that presence of NMF strengthens the barrier integrity (as judged by skin resistance measurements). Finally, high values of post-TEWL correlated with a high amount of NMF. This positive correlation implies that skin sites with higher amounts of osmotically active NMF components have a higher ability to take up water from the applied formulation, where the post-TEWL measurements mainly reflect the water absorption capacity of the skin site from the applied formulation.

### 4.6. Study Limitations

The measurements performed in this work were only conducted on healthy volunteers, which is a limitation of the study. To evaluate the suitability of the investigated sampling formulations, as well as the relevance of using the Trp/Kyn ratio as a biomarker for skin cancer diagnostics, future research initiatives should address this limitation by performing a clinical investigation on skin cancer patients. In particular, the quantities of Kyn reported in this study are only approximative estimations, since they were detected below LOQ. Overall, the analysis of Kyn was challenging due to its (as expected) low abundance on the skin surface of healthy volunteers (i.e., a hundred times lower compared to Tyr, Phe, and Trp), as well as the limited sensitivity of the analytical method.

The sampling time (i.e., 2 h) was selected based on the estimated time to reach sufficient skin hydration for efficient skin extraction [40,43], at the same time as limiting the sampling time for practical reasons. The sampling formulations potentially have different duration times for optimal sampling, which was not studied in detail.

The limited number of test subjects included in this study (*n* = 35), as well as the relatively unequal distribution of the subjects in terms of sex and age (i.e., 22 females, age range 18–64 years, mean age 35 ± 10 and 13 males, age range 26–61 years, mean age 38 ± 13), is a limitation. In particular, the underlying reason for the observed differences in terms of initial measurements of skin resistance, conductance, TEWL, and collected Trp amounts, which were observed to be different for males and females, remains uncertain.

## 5. Conclusions

The skin provides an interface from which a vast collection of disease biomarkers can be readily collected. To exploit this great potential, the development of sampling strategies and formulations with high reproducibility and good capacity to collect the biomarkers is crucial. Likewise, it is important to develop assessment protocols that allows for unbiased comparison of the extraction capacity of promising sampling formulations. This work accounts for a comprehensive evaluation protocol allowing for an unbiased comparison between four sampling formulations with different colloidal properties. The results illustrate the benefit of assessing the skin barrier’s biophysical properties with EIS and TEWL to ensure that biological differences, e.g., between females and males, or between sampling sites, are cancelled out by randomization. This is an important aspect, since it allows for an unbiased comparison of the capacities of the sampling formulations to collect biomarkers. Further, these techniques have been shown to provide useful information on the effects of different sampling formulations on the skin barrier properties, such as the hydration effect, which is helpful for identifying benefits and drawbacks of the investigated sampling formulations.

In conclusion, both the AGR and CHI hydrogels as well as the GMO and GTP liquid crystalline cubic phases can be successfully used as sampling formulations for extracting LMW analytes from skin with good reproducibility and without any observable signs of skin irritation. However, the results clearly show that the liquid crystalline cubic phases resulted in an extraction capacity that was about two times higher, as compared to the hydrogel-based formulations. Therefore, non-invasive sampling with biocompatible liquid crystalline cubic phases should be further developed and investigated as a great potential is foreseen for a wide range of biomarkers of different molecular sizes and with various physicochemical properties (e.g., hydrophilic, lipophilic, and amphiphilic biomarkers). Specifically, the assessment of specific analyte ratios represents a very reproducible approach for investigating potential biomarkers due to minimization of biological variability and discrepancies related to different sampling formulations.

## 6. Patents

Patent “Lipid patch”, ref: 21129195 [AWA-SE.SE33184.21129195], Johan Engblom, Sebastian Björklund, Maxim Morin, Skaidre Jankovskaja, and Tautgirdas Ruzgas, filed 19 October 2021.

## Figures and Tables

**Table 1:** Composition of sampling formulations.

Ingredients (wt.%)	Sampling Formulations			
	AGR	CHI	GMO	GTP
Water	98	98	38	60
Agarose	2	–	–	–
Chitosan	–	2	–	–
GMO	–	–	62	36
DOTAP	–	–	–	4
Water activity (*a*_w_)	0.997	0.993	0.999	0.999
Appearance	Stiff, clear gel	Sticky, viscous, clear gel	Viscous, clear liquid crystal	Viscous, semi-clear liquid crystal
Extraction solution	H_2_O:MeOH 80:20% (*v*/*v*)	15 mM NaN_3_ (pH 11)	H_2_O	20 mM Na_2_SO_4_

## Data Availability

Not applicable.

## Supplementary Materials

The following supporting information can be downloaded at: https://www.mdpi.com/article/10.3390/pharmaceutics14020313/s1, Figure S1: Analyte extraction from skin as a function of time; Figure S2: SAXD data of GMO and GTP; Figure S3: Representative electrical impedance data; Figure S4: Comparison of biophysical skin parameters between females and males; Figure S5: Comparison of biophysical skin parameters between skin sites; Figure S6: Comparison of extracted analyte amounts from females and males; Figure S7: Comparison of collected analyte amounts and analyte ratios from left and right arms; Figure S8: Comparison of collected analyte amounts and analyte ratios from different sampling sites on left and right arms; Figure S9: Spearman’s correlation plots between biophysical parameters measured pre and post sampling; Figure S10: Spearman’s correlation plots between the collected analyte amounts and biophysical skin parameters. Table S1: MRM transitions and chromatographic parameters; Table S2: Precision, accuracy, LOD, and LOQ of the analytical method. Table S3: Compilation of data obtained by biophysical skin measurements; Table S4: Compilation of *p*-values from statistical analyses of the data presented in Figure 2. Table S5: Incidence of application of sampling formulations on sampling sites; Table S6: Compilation of analyte amounts and analyte ratios collected by different sampling formulations. Table S7: Compilation of *p*-values from statistical analyses of the data in Figure 3; Table S8: Compilation of analyte amounts collected by different sampling formulations; Table S9: Compilation of coefficients of variation of analyte amounts and analyte ratios collected by different sampling formulations.

## References

[1] Elias P.M. (2004). The epidermal permeability barrier: From the early days at Harvard to emerging concepts. J. Investig. Dermatol..

[2] Paliwal S., Hwang B.H., Tsai K.Y., Mitragotri S. (2013). Diagnostic opportunities based on skin biomarkers. Eur. J. Pharm. Sci..

[3] Lamb R.C., Pearce J. (2021). Skin manifestations of systemic disease. Medicine.

[4] Elston D.M., Stratman E.J., Miller S.J. (2016). Skin biopsy: Biopsy issues in specific diseases. J. Am. Acad. Dermatol..

[5] Mogensen M., Jemec G.B. (2007). Diagnosis of nonmelanoma skin cancer/keratinocyte carcinoma: A review of diagnostic accuracy of nonmelanoma skin cancer diagnostic tests and technologies. Dermatol. Surg..

[6] Abhishek K., Khunger N. (2015). Complications of skin biopsy. J. Cutan. Aesthetic Surg..

[7] Narayanamurthy V., Padmapriya P., Noorasafrin A., Pooja B., Hema K., Khan A.Y.F., Nithyakalyani K., Samsuri F. (2018). Skin cancer detection using non-invasive techniques. RSC Adv..

[8] Rayess H.M., Gupta A., Svider P.F., Raza S.N., Shkoukani M., Zuliani G.F., Carron M.A. (2017). A critical analysis of melanoma malpractice litigation: Should we biopsy everything?. Laryngoscope.

[9] Blank I.H., Moloney J., Emslie A.G., Simon I., Apt C. (1984). The diffusion of water across the stratum corneum as a function of its water content. J. Investig. Dermatol..

[10] Scheuplein R.J., Blank I.H. (1971). Permeability of the skin. Physiol. Rev..

[11] Ogawa-Fuse C., Morisaki N., Shima K., Hotta M., Sugata K., Ichihashi T., Oguri M., Yoshida O., Fujimura T. (2019). Impact of water exposure on skin barrier permeability and ultrastructure. Contact Dermat..

[12] Björklund S., Engblom J., Thuresson K., Sparr E. (2010). A water gradient can be used to regulate drug transport across skin. J. Control. Release.

[13] Albér C., Brandner B.D., Björklund S., Billsten P., Corkery R.W., Engblom J. (2013). Effects of water gradients and use of urea on skin ultrastructure evaluated by confocal Raman microspectroscopy. BBA—Biomembr..

[14] Santos L.F., Correia I.J., Silva A.S., Mano J.F. (2018). Biomaterials for drug delivery patches. Eur. J. Pharm. Sci..

[15] Hoffman A.S. (2012). Hydrogels for biomedical applications. Adv. Drug Deliv. Rev..

[16] Dutkiewicz E.P., Lin J.D., Tseng T.W., Wang Y.S., Urban P.L. (2014). Hydrogel micropatches for sampling and profiling skin metabolites. Anal. Chem..

[17] Dutkiewicz E.P., Hsieh K.T., Urban P.L., Chiu H.Y. (2020). Temporal Correlations of Skin and Blood Metabolites with Clinical Outcomes of Biologic Therapy in Psoriasis. J. Appl. Lab. Med..

[18] Qiu Y., Park K. (2012). Environment-sensitive hydrogels for drug delivery. Adv. Drug Deliv. Rev..

[19] Hoare T.R., Kohane D.S. (2008). Hydrogels in drug delivery: Progress and challenges. Polymer.

[20] Luzzati V. (1997). Biological significance of lipid polymorphism: The cubic phases—Commentary. Curr. Opin. Struct. Biol..

[21] Larsson K. (1989). Cubic Lipid-Water Phases—Structures and Biomembrane Aspects. J. Phys. Chem..

[22] Barauskas J., Landh T. (2003). Phase behavior of the phytantriol/water system. Langmuir.

[23] Clogston J., Caffrey M. (2005). Controlling release from the lipidic cubic phase. Amino acids, peptides, proteins and nucleic acids. J. Control. Release.

[24] Bender J., Ericson M.B., Merclin N., Iani V., Rosen A., Engstrom S., Moan J. (2005). Lipid cubic phases for improved topical drug delivery in photodynamic therapy. J. Control. Release.

[25] Lopes L.B., Lopes J.L., Oliveira D.C., Thomazini J.A., Garcia M.T., Fantini M.C., Collett J.H., Bentley M.V. (2006). Liquid crystalline phases of monoolein and water for topical delivery of cyclosporin A: Characterization and study of in vitro and in vivo delivery. Eur. J. Pharm. Biopharm..

[26] Angelova A., Angelov B., Mutafchieva R., Lesieur S., Couvreur P. (2011). Self-assembled multicompartment liquid crystalline lipid carriers for protein, peptide, and nucleic acid drug delivery. Acc. Chem. Res..

[27] Yu X., Jin Y., Du L., Sun M., Wang J., Li Q., Zhang X., Gao Z., Ding P. (2018). Transdermal Cubic Phases of Metformin Hydrochloride: In Silico and in Vitro Studies of Delivery Mechanisms. Mol. Pharm..

[28] Mohammady S.Z., Pouzot M., Mezzenga R. (2009). Oleoylethanolamide-based lyotropic liquid crystals as vehicles for delivery of amino acids in aqueous environment. Biophys. J..

[29] Landau E.M., Rummel G., CowanJacob S.W., Rosenbusch J.P. (1997). Crystallization of a polar protein and small molecules from the aqueous compartment of lipidic cubic phases. J. Phys. Chem. B.

[30] Rummel G., Hardmeyer A., Widmer C., Chiu M.L., Nollert P., Locher K.P., Pedruzzi I.I., Landau E.M., Rosenbusch J.P. (1998). Lipidic Cubic Phases: New Matrices for the Three-Dimensional Crystallization of Membrane Proteins. J. Struct. Biol..

[31] Caffrey M., Cherezov V. (2009). Crystallizing membrane proteins using lipidic mesophases. Nat. Protoc..

[32] Scott I.R., Harding C.R. (1986). Filaggrin breakdown to water binding-compounds during development of the rat stratum-corneum is controlled by the water activity of the environment. Dev. Biol..

[33] Jankovskaja S., Engblom J., Rezeli M., Marko-Varga G., Ruzgas T., Björklund S. (2021). Non-invasive skin sampling of tryptophan/kynurenine ratio in vitro towards a skin cancer biomarker. Sci. Rep..

[34] Björklund S., Andersson J.M., Pham Q.D., Nowacka A., Topgaard D., Sparr E. (2014). Stratum corneum molecular mobility in the presence of natural moisturizers. Soft Matter.

[35] Björklund S., Engblom J., Thuresson K., Sparr E. (2013). Glycerol and urea can be used to increase skin permeability in reduced hydration conditions. Eur. J. Pharm. Sci..

[36] Rawlings A.V., Scott I.R., Harding C.R., Bowser P.A. (1994). Stratum-Corneum Moisturization at the Molecular-Level. J. Investig. Dermatol..

[37] Li H., Bullock K., Gurjao C., Braun D., Shukla S.A., Bosse D., Lalani A.A., Gopal S., Jin C., Horak C. (2019). Metabolomic adaptations and correlates of survival to immune checkpoint blockade. Nat. Commun..

[38] Suzuki Y., Suda T., Furuhashi K., Suzuki M., Fujie M., Hahimoto D., Nakamura Y., Inui N., Nakamura H., Chida K. (2010). Increased serum kynurenine/tryptophan ratio correlates with disease progression in lung cancer. Lung Cancer.

[39] Weinlich G., Murr C., Richardsen L., Winkler C., Fuchs D. (2007). Decreased serum tryptophan concentration predicts poor prognosis in malignant melanoma patients. Dermatology.

[40] Morin M., Ruzgas T., Svedenhag P., Anderson C.D., Ollmar S., Engblom J., Björklund S. (2020). Skin hydration dynamics investigated by electrical impedance techniques in vivo and in vitro. Sci. Rep..

[41] Rinaldi A.O., Korsfeldt A., Ward S., Burla D., Dreher A., Gautschi M., Stolpe B., Tan G., Bersuch E., Melin D. (2021). Electrical impedance spectroscopy for the characterization of skin barrier in atopic dermatitis. Allergy.

[42] Team R. (2020). RStudio: Integrated Development Environment for R. RStudio.

[43] Björklund S., Ruzgas T., Nowacka A., Dahi I., Topgaard D., Sparr E., Engblom J. (2013). Skin membrane electrical impedance properties under the influence of a varying water gradient. Biophys. J..

[44] Nicander I., Nyren M., Emtestam L., Ollmar S. (1997). Baseline electrical impedance measurements at various skin sites—Related to age and sex. Skin Res. Technol..

[45] Luebberding S., Krueger N., Kerscher M. (2013). Skin physiology in men and women: In vivo evaluation of 300 people including TEWL, SC hydration, sebum content and skin surface pH. Int. J. Cosmet. Sci..

[46] Lammintausta K., Maibach H.I., Wilson D. (1987). Irritant reactivity in males and females. Contact Dermat..

[47] Tupker R.A., Coenraads P.J., Pinnagoda J., Nater J.P. (1989). Baseline transepidermal water loss (TEWL) as a prediction of susceptibility to sodium lauryl sulphate. Contact Dermat..

[48] Darlenski R., Fluhr J.W. (2012). Influence of skin type, race, sex, and anatomic location on epidermal barrier function. Clin. Dermatol..

[49] Zabara A., Mezzenga R. (2014). Controlling molecular transport and sustained drug release in lipid-based liquid crystalline mesophases. J. Control. Release.

[50] Pham Q.D., Björklund S., Engblom J., Topgaard D., Sparr E. (2016). Chemical penetration enhancers in stratum corneum—Relation between molecular effects and barrier function. J. Control. Release.

[51] Duangjit S., Pamornpathomkul B., Opanasopit P., Rojanarata T., Obata Y., Takayama K., Ngawhirunpat T. (2014). Role of the charge, carbon chain length, and content of surfactant on the skin penetration of meloxicam-loaded liposomes. Int. J. Nanomed..

[52] Sylvestre J.P., Bouissou C.C., Guy R.H., Delgado-Charro M.B. (2010). Extraction and quantification of amino acids in human stratum corneum in vivo. Br. J. Dermatol..

[53] Stoffers K.M., Cronkright A.A., Huggins G.S., Baleja J.D. (2020). Noninvasive Epidermal Metabolite Profiling. Anal. Chem..

[54] Wirthgen E., Hoeflich A. (2015). Endotoxin-Induced Tryptophan Degradation along the Kynurenine Pathway: The Role of Indolamine 2,3-Dioxygenase and Aryl Hydrocarbon Receptor-Mediated Immunosuppressive Effects in Endotoxin Tolerance and Cancer and Its Implications for Immunoparalysis. J. Amino Acids.

[55] Maeno K., Shida Y., Shimada H. (2017). Direct quantitative analysis of the natural moisturizing factor (NMF) in the stratum corneum by direct analysis in real time mass spectrometry (DART-MS). Anal. Methods.

[56] Madison K.C. (2003). Barrier function of the skin: “La Raison d’Etre” of the epidermis. J. Investig. Dermatol..

[57] Karande P., Jain A., Mitragotri S. (2006). Relationships between skin’s electrical impedance and permeability in the presence of chemical enhancers. J. Control. Release.

[58] Rinaldi A.O., Morita H., Wawrzyniak P., Dreher A., Grant S., Svedenhag P., Akdis C.A. (2019). Direct assessment of skin epithelial barrier by electrical impedance spectroscopy. Allergy.

