# Supplementary material for: Hydrogels and Cubic Liquid Crystals for Non-Invasive Sampling of Low-Molecular-Weight Biomarkers—An Explorative In Vivo Study
Source: Pharmaceutics. 2022 Jan 28;14(2):313. doi: 10.3390/pharmaceutics14020313 (PMC8879558; doi:10.3390/pharmaceutics14020313)
Supplement: Supplementary file 1 [file pharmaceutics-14-00313-s001.zip › pharmaceutics-1506931-supplementary.pdf]

Hydrogels and cubic liquid crystals for non-invasive sampling of low molecular weight biomarkers – an explorative *in vivo* study

Maxim Morin [1,2,†], Skaidre Jankovskaja [1,2,†], Tautgirdas Ruzgas [1,2], Joakim Henricson [3,4], Chris D. Anderson [5], Anders Brinte [6], Johan Engblom [1,2] and Sebastian Björklund [1,2,*]

[1] Department of Biomedical Sciences, Faculty of Health and Society, Malmö University, SE-205 06 Malmö, Sweden
[2] Biofilms – Research Center for Biointerfaces, Malmö University, SE-205 06 Malmö, Sweden
[3] Division of Clinical Chemistry and Pharmacology, Department of Biomedical and Clinical Sciences, Faculty of Health Sciences, Linköping University, SE-581 83 Linköping, Sweden
[4] Department of Emergency Medicine, Local Health Care Services in Central Östergötland, SE-581 85 Linköping, Sweden
[5] Division of Cell Biology, Department of Biomedical and Clinical Sciences, Faculty of Health Sciences, Linköping University, SE-581 83 Linköping, Sweden
[6] ImaGene-iT, Medicon Village, SE-223 63, Lund, Sweden
† Co-first author, these authors contributed equally to this work
* Correspondence: sebastian.bjorklund@mau.se

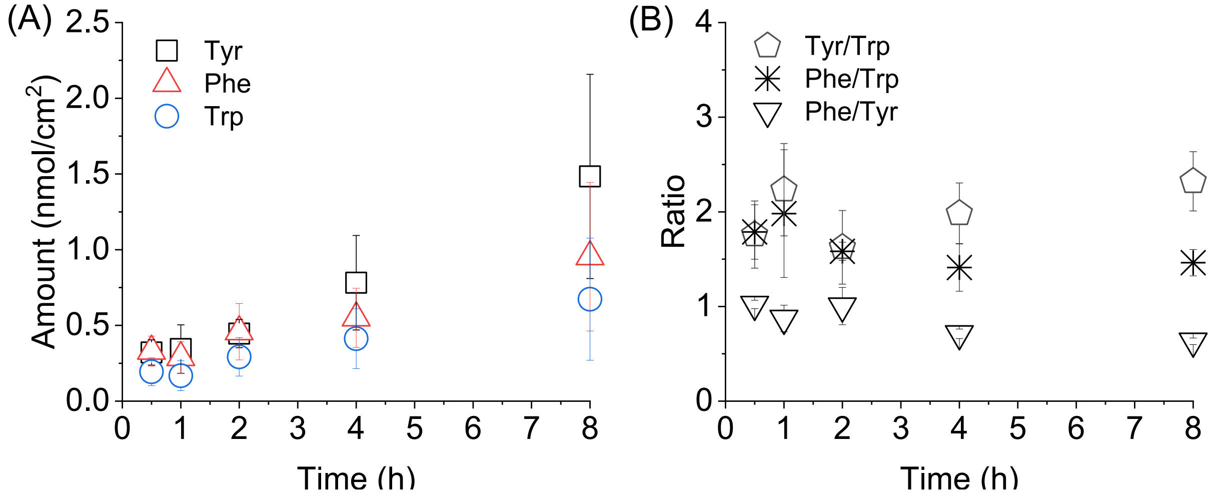

Figure S1. Skin extraction of analytes as a function of time. Data show cumulative amount (A) of tyrosine (Tyr), phenylalanine (Phe), and tryptophan (Trp) and their corresponding ratios (B) as a function of time. CHI (chitosan) hydrogel was used as sampling formulation. For each time point, the hydrogel was applied on the skin surface at 3 adjacent locations on the same individual. The Tyr/Trp and Phe/Trp ratios were not statistically different over the different sampling time. The Phe/Tyr ratio was significantly higher for samples collected over 0.5h, as compared to 4h (p=0.045) and 8h (p=0.012); Phe/Tyr ratio determined in samples collected over 2h was significantly higher compared to 8 h (p=0.016). Comparison between amounts and ratios was done by performing one-way ANOVA with multiple testing (Tukey test).

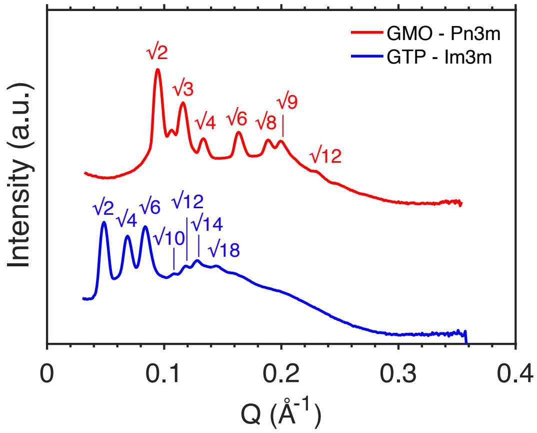

Figure S2. Small angle X-ray diffraction (SAXD) results from the cubic phases, confirming the existence of Pn3m cubic lattice for GMO (red) and Im3m cubic lattice for GTP (blue). The SAXD data were collected with a Xeuss 3.0 SAXS/WAXS laboratory-based instrument (Xenocs, France) at Malmö University (Malmö, Sweden). The X-ray beam is generated by Cu K_α radiation source (λ = 1.541 Å). All samples were measured in the ambient at 25 °C, with a temperature-controlled Peltier gel-holder stage using an O-ring as a spacer between two Kapton films (DuPontTM Kapton®, 0.013 mm thickness, Goodfellow, England). The system operates with a Pilatus3 R 300K hybrid photon counting detector with a sample-to-detector distance (STDD) of 800 mm and 1700 mm. This STDD range covers a q-range between 0.0002 ≤ q (Å-1) ≤ 0.36, where q is the scattering vector defined as $q = (4\pi/\lambda)\sin(\theta/2)$ where θ is the scattering angle. One-dimensional (1D) data was obtained by azimuthal averaging of the 2D-diffraction pattern and the scattering intensity was corrected for background scattering and normalized to direct beam. The normal exposure time was 30 minutes for each sample.

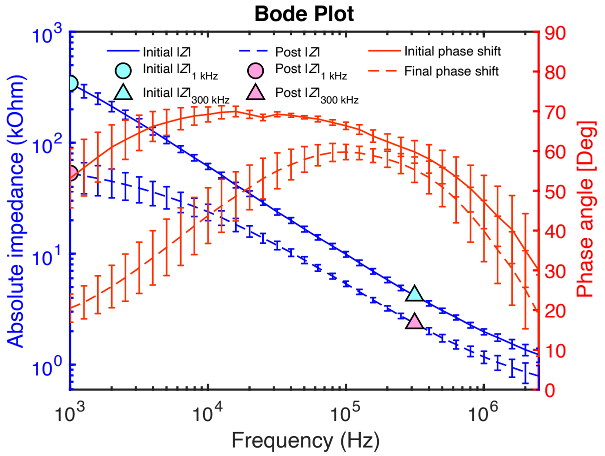

Figure S3. Representative impedance data obtained pre and post sampling of the same skin site (in this example, CHI was applied on the skin site for 2h). The data is presented in the form of a Bode plot, where the absolute impedance |Z| (blue) and the phase angle (red) are plotted as a function of frequency. The initial data are shown as solid lines, while the post sampling data are shown as dashed line. The filled circles at 1 kHz show the data points defined as the skin resistance (i.e., $|Z|_{1kHz}$). The filled triangles show the data used to calculate the skin conductance (i.e., $1/|Z|_{315kHz}$). Cyan data points represent initial data, while pink data points represent post sampling data.

Table S1. Retention times (RT), molecular weight (MW), selected precursor and product ions for multiple reaction monitoring (MRM), and collision energies (CE) for each analyte.

Analyte	RT (min)	MW (Da)	Precursor ion	Precursor (m/z)	Product (m/z)	CE (eV)
L-Tyrosine	7.3	181.19	$[M+H]^+$	182.1	165.3	10
				182.1	136.1	10
$[^2H_4]$-L-Tyrosine		185.21	$[M+H]^+$	186.3	140.4	10
				186.3	169.3	10
L-Kynurenine	8.6	208.22	$[M+H]^+$	209.3	146.2	30
				209.3	192.3	10
$[^{13}C_6]$-L-Kynurenine		214.17	$[M+H]^+$	215.3	152.3	30
				215.3	198.3	10
L-Phenylalanine	9.3	165.19	$[M+H]^+$	166.3	102.1	20
				166.3	120.2	20
$[^2H_2]$-L-Phenylalanine		167.20	$[M+H]^+$	168.3	104.3	20
				168.3	122.3	20
L-Tryptophan	10.5	204.23	$[M+H]^+$	205.3	91.2	35
				205.3	118.3	30
$[^{13}C_{11}, ^{15}N_2]$-L-Tryptophan		217.13	$[M+H]^+$	218.3	98.2	35
				218.3	127.3	30

Table S2A. Precision of quantification of the analytical method. Calibration solutions were prepared for Tyr, Phe, Trp, and Kyn with addition of corresponding isotopically labelled spike (IS) at 0.25 μM. The IS/analyte peak area ratios were determined over three consecutive days. The precision is defined as CV = SD/Mean x 100%. Values below LOQ (see Table S2C) were excluded (n. a.).

Concentration (μM)	Concentration (μM)	CV(%)			
Trp, Phe, Kyn	Tyr	Trp	Phe	Kyn	Tyr
0.0625	0.25	n. a.	n. a.	n. a.	n. a.
0.125	0.5	n. a.	n. a.	n. a.	n. a.
0.25	1	30	8	44	3
0.5	2	5	15	16	11
1	4	8	11	16	27
2	8	27	26	13	22

Table S2B. Accuracy of the analytical method. Calibration solutions were prepared for Tyr, Phe, Trp, and Kyn with addition of corresponding isotopically labelled spike (IS) at 0.25 μM. The IS/analyte peak area ratios were determined over three consecutive days. Accuracy is given as the recovery of the analyte with respect to the IS where full recovery is defined as 100%. Values below LOQ (see Table S2C) were excluded (n. a.).

Concentration (μM)		Accuracy (%)				SD (%)			
Trp, Phe, Kyn	Tyr	Trp	Phe	Kyn	Tyr	Trp	Phe	Kyn	Tyr
0.0625	0.25	n. a.	n. a.	n. a.	n. a.	n. a.	n. a.	n. a.	n. a.
0.125	0.5	n. a.	n. a.	n. a.	n. a.	n. a.	n. a.	n. a.	n. a.
0.25	1	96	81	84	123	28	6	37	3
0.5	2	75	70	83	140	4	11	13	16
1	4	76	74	85	124	6	8	13	34
2	8	84	82	90	139	23	21	12	30

Table S2C. Limit of detection (LOD) and quantification (LOQ) of the analytical method. A linear regression analysis was performed by plotting the measured concentration (n=3) as a function of theoretical concentration (i.e., measured concentration = slope × theoretical concentration + y-intercept). The LOD and LOQ were calculated as follows; LOD = 3.3σ/slope and LOQ as LOQ = 10σ/slope, where σ is the standard error of the y-intercept from the regression analysis.

Parameter	Concentration (μM)			
	Trp	Phe	Kyn	Tyr
LOD	0.08	0.07	0.05	0.28
LOQ	0.23	0.22	0.15	0.86

Table S3. Compilation of data obtained by biophysical skin measurements. Skin resistance (i.e., $|Z|_{1kHz}$), conductance (i.e., $1/|Z|_{315kHz}$), and TEWL measured pre/post sampling with four different formulations (AGR, CHI, GMO, and GTP). The EI measurements were performed on the right arm (sites R1-R4,) while TEWL was measured on both arms (i.e., sites R1-R4 and L1-L4). Data are shown as mean ± SD.

Factor	|Z| at 1 kHz (kOhm)		Conductance (μSi)		TEWL, right (g/m²h)		TEWL, left (g/m²h)	
	Pre	Post	Pre	Post	Pre	Post	Pre	Post
Overall	402.1 ± 246.3 (n = 131)	295.5 ± 132.0 (n = 131)	174.3 ± 66.6 (n = 140)	248.9 ± 64.5 (n = 135)	9.9 ± 1.8 (n = 137)	39.7 ± 7.2 (n = 133)	9.5 ± 1.6 (n = 132)	40.6 ± 8.8 (n = 134)
Sex	Pre	Post	Pre	Post	Pre	Post	Pre	Post
Female	445.5 ± 269.4 n = 80	301.9 ± 142.7 n = 84	165.5 ± 71.5 n = 88	242.3 ± 65.0 n = 83	9.6 ± 1.9 n = 85	39.7 ± 7.5 n = 84	9.0 ± 1.7 n = 80	40.0 ± 8.6 n = 84
Male	334.1 ± 187.7 n = 51	285.0 ± 113.0 n = 52	189.1 ± 54.9 n = 52	259.4 ± 62.8 n = 52	10.4 ± 1.4 n = 52	39.6 ± 6.6 n = 49	10.2 ± 1.2 n = 52	42.0 ± 9.5 n = 51
Site	Pre	Post*	Pre	Post*	Pre	Post*	Pre	Post*
R1 (L1)	261.3 ± 123.1 n = 31	244.8 ± 116.2 n = 33	182.2 ± 63.8 n = 35	272.6 ± 78.7 n = 34	10.1 ± 2.0 n = 34	40.9 ± 7.4 n = 34	9.6 ± 1.7 n = 35	42.8 ± 10.3 n = 35
R2 (L2)	353.5 ± 219.6 n = 31	250.7 ± 134.7 n = 34	169.7 ± 70.0 n = 35	237.0 ± 60.8 n = 33	10.0 ± 1.7 n = 34	40.2 ± 7.1 n = 34	9.4 ± 1.9 n = 35	37.6 ± 7.0 n = 31
R3 (L3)	467.6 ± 239.7 n = 32	339.9 ± 132.1 n = 34	159.8 ± 61.6 n = 35	237.2 ± 49.1 n = 33	9.6 ± 1.4 n = 32	38.9 ± 7.6 n = 33	9.6 ± 1.7 n = 34	40.5 ± 7.2 n = 34
R4 (L4)	450.7 ± 251.8 n = 33	285.2 ± 106.6 n = 33	179.4 ± 70.1 n = 35	252.3 ± 57.2 n = 34	10.0 ± 1.4 n = 32	38.7 ± 6.8 n = 32	9.8 ± 1.7 n = 33	39.9 ± 8.2 n = 32
Formulation	Pre	Post	Pre	Post	Pre	Post	Pre	Post
AGR	366.4 ± 215.8 n = 32	354.3 ± 125.8 n = 34	175.7 ± 67.7 n = 35	222.6 ± 38.6 n = 30	9.8 ± 1.7 n = 34	39.3 ± 6.3 n = 34	9.7 ± 1.8 n = 33	37.6 ± 4.7 n = 34
CHI	397.9 ± 228.2 n = 33	245.0 ± 132.4 n = 34	177.0 ± 67.1 n = 35	247.9 ± 59.8 n = 31	9.5 ± 1.9 n = 34	35.9 ± 5.0 n = 35	9.7 ± 1.8 n = 34	35.3 ± 6.0 n = 35
GMO	372.5 ± 215.3 n = 30	340.1 ± 140.2 n = 34	170.6 ± 67.1 n = 35	239.6 ± 70.7 n = 34	10.1 ± 2.0 n = 35	39.1 ± 5.7 n = 35	9.3 ± 1.5 n = 34	41.1 ± 7.7 n = 35
GTP	405.1 ± 244.8 n = 33	242.4 ± 84.1 n = 34	173.7 ± 67.1 n = 35	265.8 ± 51.5 n = 34	10.1 ± 1.5 n = 35	48.3 ± 9.0 n = 31	9.6 ± 1.9 n = 35	51.0 ± 10.7 n = 32

*These values should be interpreted with caution since the formulations were randomly distributed between the sampling sites.

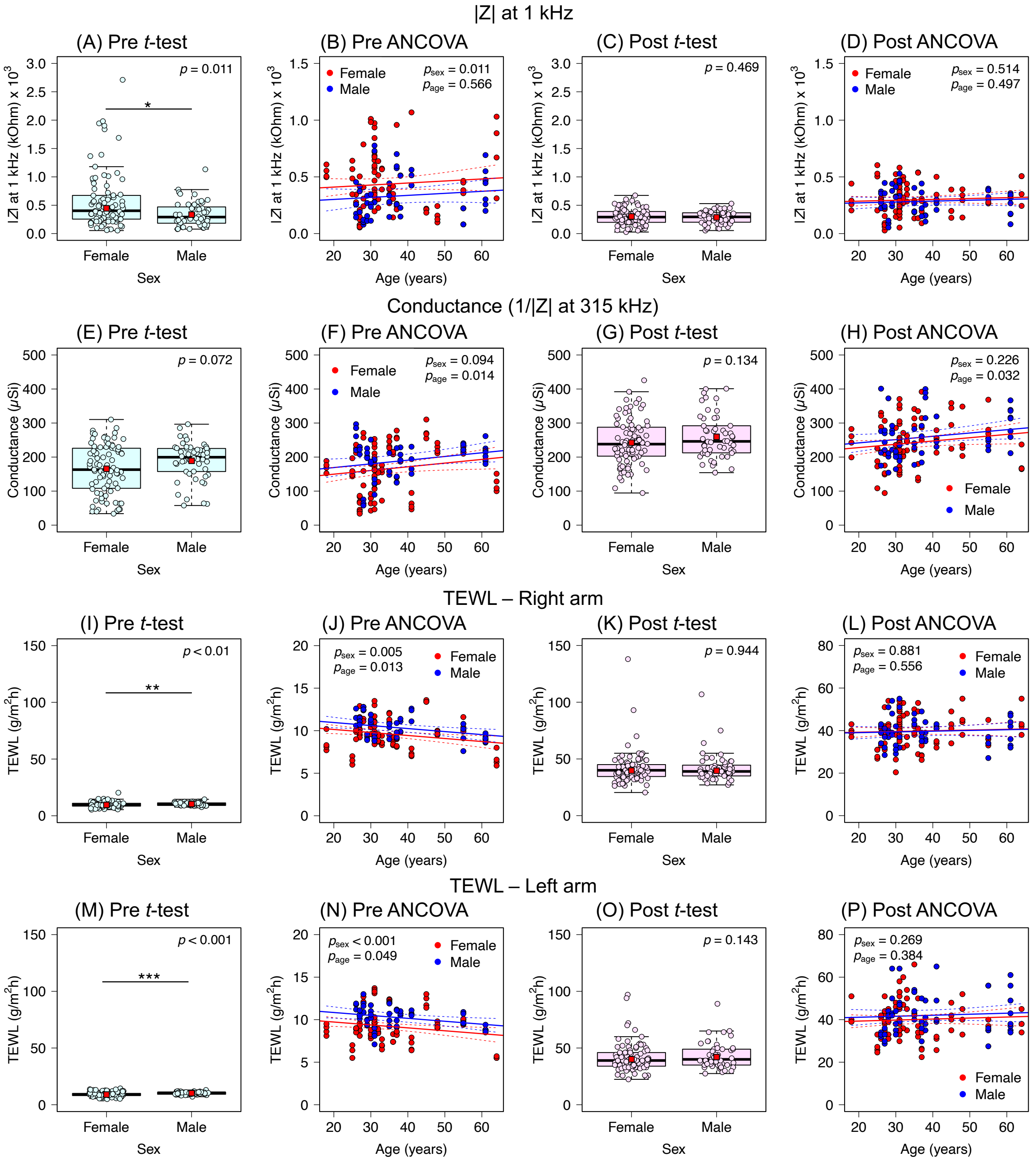

Figure S4. Comparison of biophysical skin parameters between females and males. The results show pre and post skin resistance (i.e., $|Z|_{1kHz}$, A-D), conductance (i.e., $1/|Z|_{315kHz}$, E-H), and TEWL (right arms, I-L and left arms, M-P) performed on females and males. Panels (A, E, I, M) and (C, G, K, O) show t-tests, while panels (B, F, J, N) and (D, H, L, P) show ANCOVA analyses taking into account age as covariable. The significance levels used were: $*p < 0.05$, $**p < 0.01$, $***p < 0.001$.

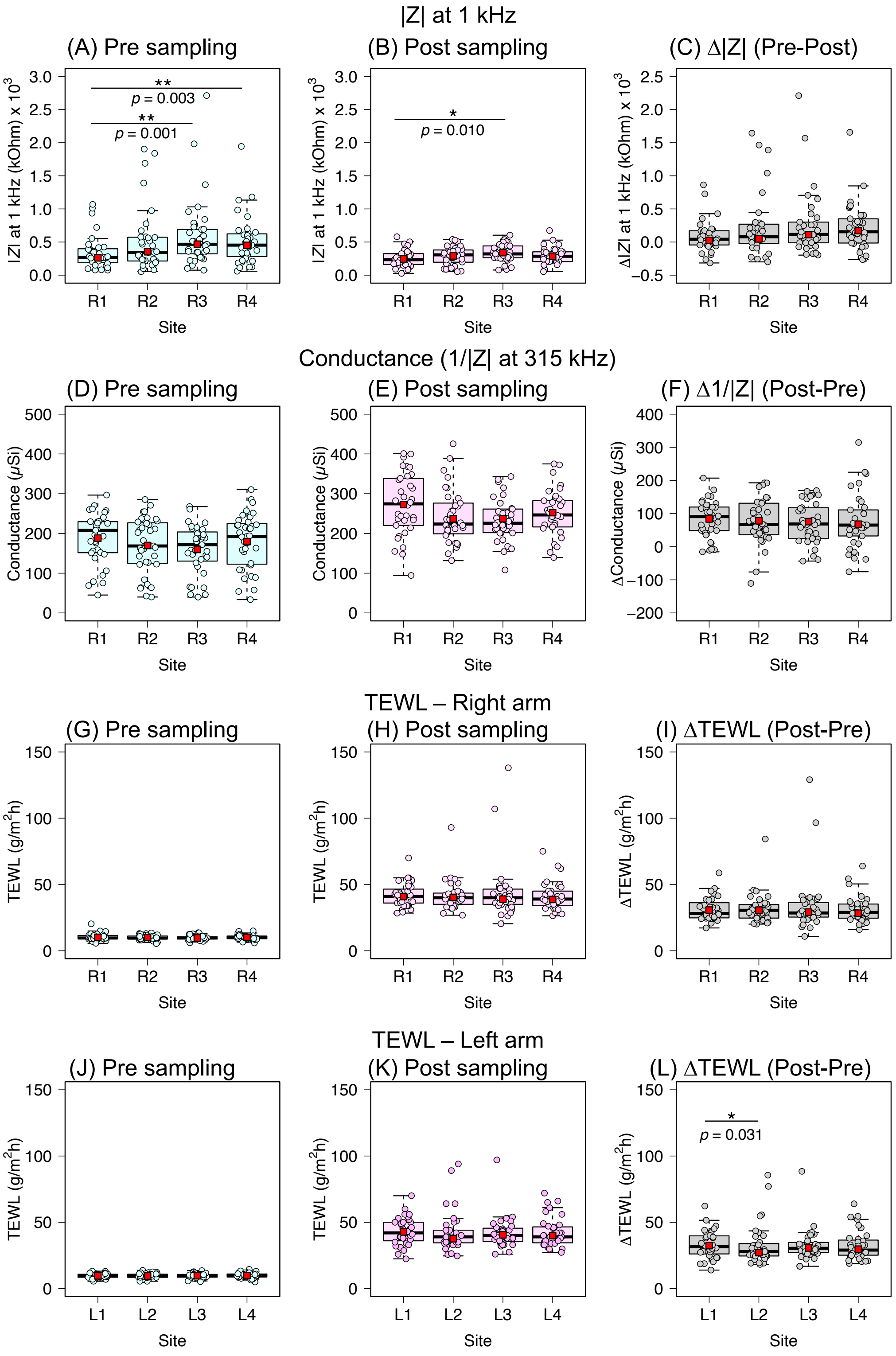

Figure S5. Comparison of biophysical skin parameters between skin sites. The results show pre (left panel, A, D, G, J) and post (middle panel, B, E, H, K), as well the difference between pre and post data (right panel, C, F, I, L) from different sampling sites. Skin resistance values represent |Z| at 1 kHz (i.e., $|Z|_{1kHz}$) and conductance values represent 1/|Z| at 300 kHz (i.e., $1/|Z|_{315kHz}$). The significance levels used were: *$p < 0.05$, **$p < 0.01$, ***$p < 0.001$.

Table S4. Compilation of *p*-values from the statistical analyses (one-way ANOVA with multiple testing, Tukey test) of the skin barrier biophysical data presented in Figure 2 in the main article. The significance levels used were: $*p < 0.05$, $**p < 0.01$, $***p < 0.001$.

	***p*-value**											
	**|Z| at 1 kHz**			**Conductance (1/|Z| at 315 kHz)**			**TEWL (Right arm)**			**TEWL (Left arm)**		
	**Pre**	**Post**	**Δ**	**Pre**	**Post**	**Δ**	**Pre**	**Post**	**Δ**	**Pre**	**Post**	**Δ**
CHI - AGR	0.943 (n.s.)	0.002 (**)	0.096 (n.s.)	1.000 (n.s.)	0.309 (n.s.)	0.615 (n.s.)	0.924 (n.s.)	0.157 (n.s.)	0.145 (n.s.)	1.000 (n.s.)	0.576 (n.s.)	0.577 (n.s.)
GMO - AGR	1.000 (n.s.)	0.963 (n.s.)	0.999 (n.s.)	0.989 (n.s.)	0.632 (n.s.)	0.873 (n.s.)	0.908 (n.s.)	0.999 (n.s.)	1.000 (n.s.)	0.825 (n.s.)	0.225 (n.s.)	0.217 (n.s.)
GTP - AGR	0.901 (n.s.)	0.001 (**)	0.263 (n.s.)	0.999 (n.s.)	0.015 (*)	0.048 (*)	0.932 (n.s.)	< 0.001 (***)	< 0.001 (***)	1.000 (n.s.)	< 0.001 (***)	< 0.001 (***)
GMO - CHI	0.971 (n.s.)	0.009 (**)	0.151 (n.s.)	0.978 (n.s.)	0.936 (n.s.)	0.957 (n.s.)	0.567 (n.s.)	0.193 (n.s.)	0.166 (n.s.)	0.784 (n.s.)	0.008 (**)	0.006 (**)
GTP - CHI	0.999 (n.s.)	1.000 (n.s.)	0.963 (n.s.)	0.997 (n.s.)	0.586 (n.s.)	0.533 (n.s.)	0.613 (n.s.)	< 0.001 (***)	< 0.001 (***)	0.999 (n.s.)	< 0.001 (***)	< 0.001 (***)
GTP - GMO	0.941 (n.s.)	0.007 (**)	0.361 (n.s.)	0.998 (n.s.)	0.234 (n.s.)	0.220 (n.s.)	1.000 (n.s.)	< 0.001 (***)	< 0.001 (***)	0.856 (n.s.)	< 0.001 (***)	< 0.001 (***)

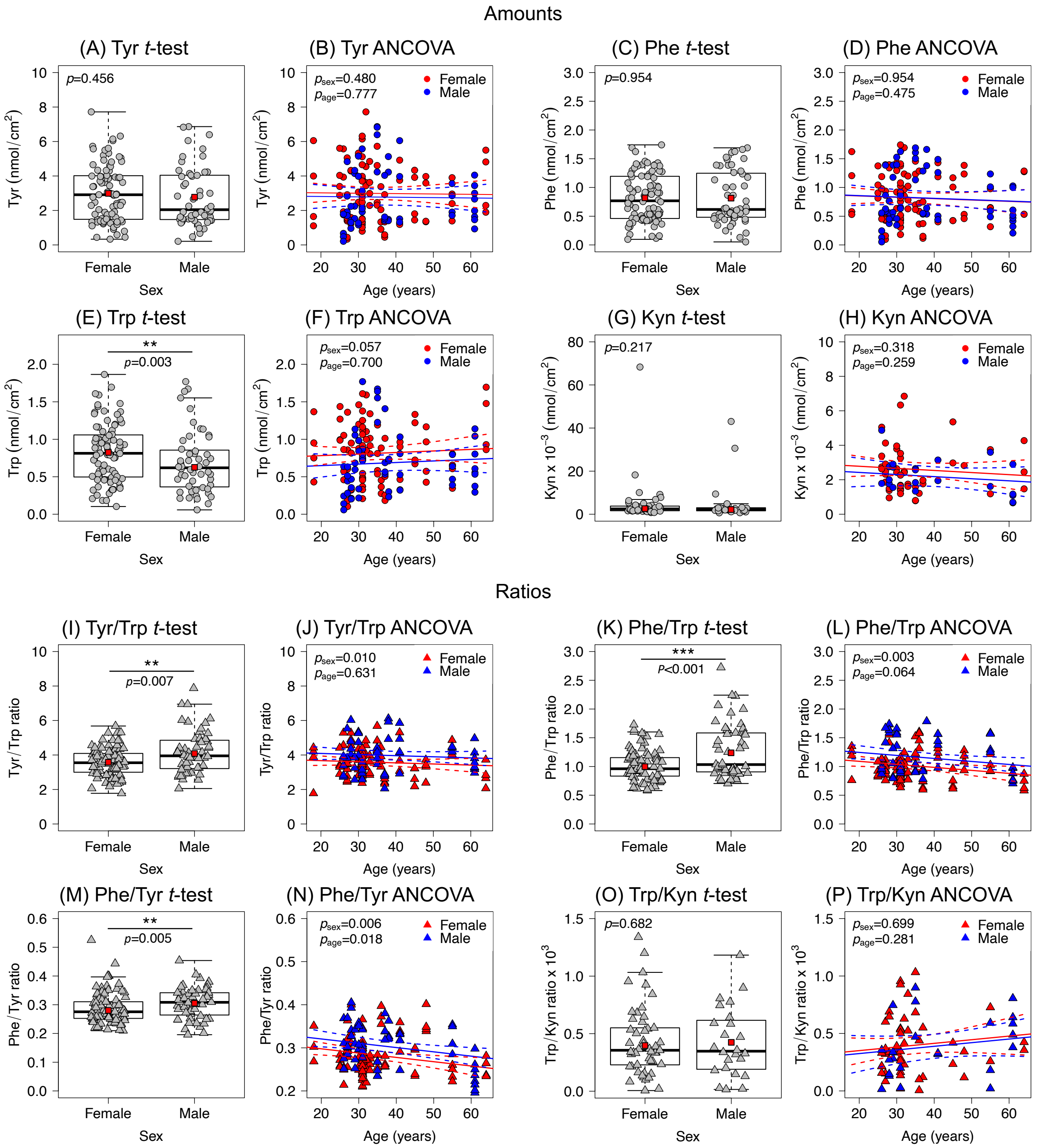

Figure S6. Comparison of extracted analyte amounts from females and males. The data show the absolute quantity of Tyr (A), Phe (C), Trp (E), and Kyn (G) and their corresponding ratios Tyr/Trp (I), Phe/Trp (K), Phe/Tyr (M), and Trp/Kyn (O) determined in the samples collected from female and male forearms and statistically evaluated with *t*-tests. ANCOVA analyses, taking into account age as covariable, of the corresponding data are presented in panels (B,F,J,N) and (D,H,L,P). The significance levels used were: $^{*}p < 0.05$, $^{**}p < 0.01$, $^{***}p < 0.001$.

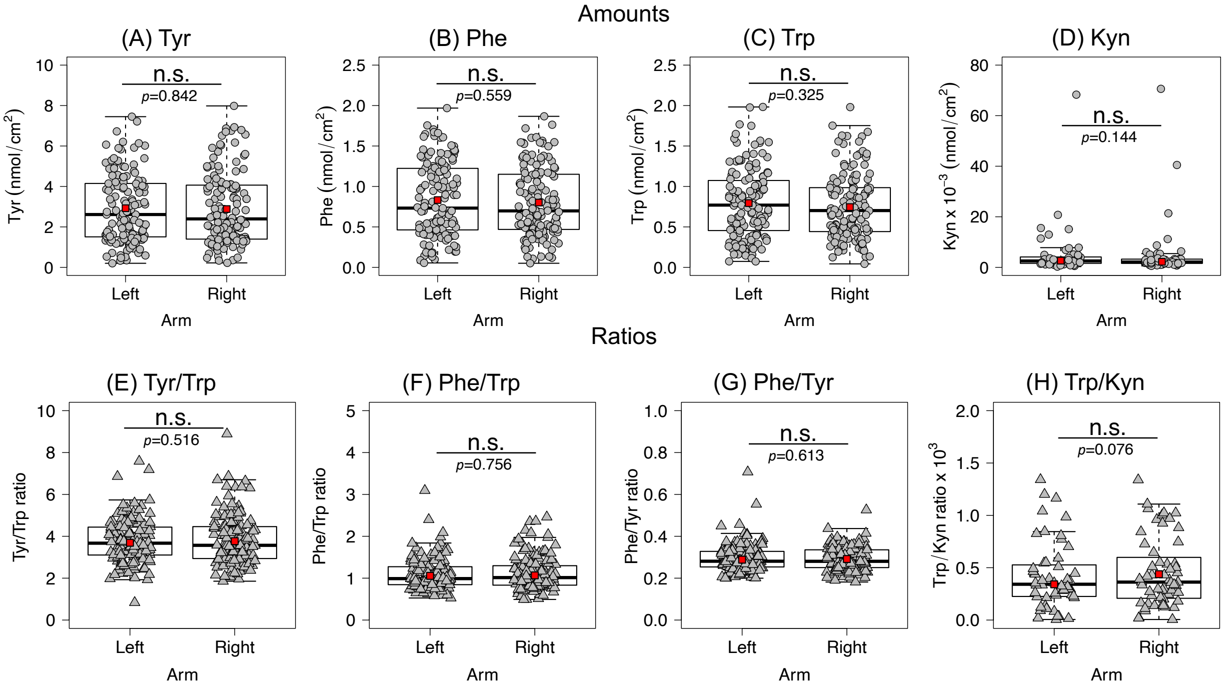

Figure S7. Comparison of collected analyte amounts and analyte ratios from left and right arms. The data show amounts of Tyr (A), Phe (B), Trp (C), and Kyn (D) and their corresponding ratios Tyr/Trp (E), Phe/Trp (F), Phe/Tyr (G), and Trp/Kyn (H). The significance levels used were: $^{*}p < 0.05$, $^{**}p < 0.01$, $^{***}p < 0.001$.

Table S5. Incidence of application of sampling formulations on sampling sites (see Figure 1 in the main article).

Site	Incidence of application of the sampling formulation			
	AGR	CHI	GMO	GTP
L1 / R1	9 (26%)	7 (20%)	13 (37%)	6 (17%)
L2 / R2	9 (26%)	12 (34%)	6 (17%)	8 (23%)
L3 / R3	11 (31%)	5 (14%)	9 (26%)	10 (29%)
L4 / R4	6 (17%)	11 (31%)	7 (20%)	11 (31%)

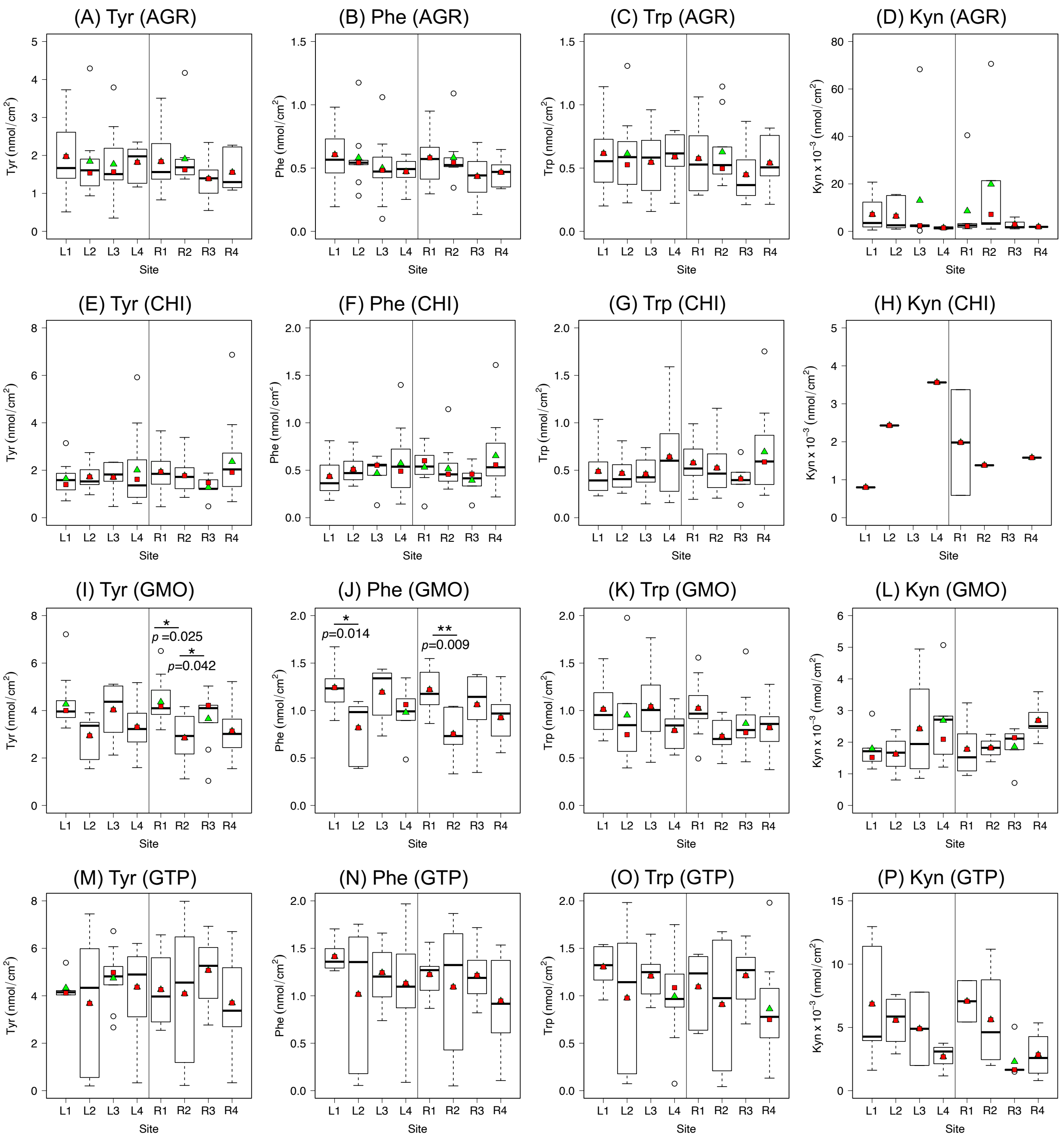

Figure S8. Comparison of collected analyte amounts and analyte ratios from different sampling sites on left (L1, L2, L3, L4) and right (R1, R2, R3, R4) arms. The results show analyte amounts collected by AGR (A,B,C,D), CHI (E,F,G,H), GMO (I,J,K,L), and GTP (M,N,O,P) as sampling formulations. The boundaries of the boxplot represent the first and third quartiles, the thick bar shows the median value and the vertical line reports the range of the values observed. Data points above or below maxima/minima (vertical line of the boxplot) are considered as outliers. Mean values (green triangles) were calculated based on untreated data, whereas mean values (red squares) were calculated after statistical removal of outliers. The number of the measurements performed on each site can be found in Table S5. Statistical analysis was performed on the data sets after removing outliers. Pairwise comparison between the quantity of analytes collected from different sites, but from the same arm, was done by performing one-way ANOVA with Tukey test. The significance levels used were: $*p < 0.05$, $**p < 0.01$, $***p < 0.001$.

Table S6. Compilation of analyte amounts (A) and their corresponding ratios (B) collected by different sampling formulations. Statistical parameters such as mean value, standard deviation and median, were calculated from the raw data (RD) and the raw data after removal of statistical outliers (OR).

(A) Amounts

Sampling formulation	Statistical parameter	Amount (nmol/cm²)			
		Tyr	Phe	Trp	Kyn (x10³)
AGR	Mean RD ± SD RD	1.8 ± 0.8	0.5 ± 0.2	0.6 ± 0.3	9.4± 17.3
	Mean OR ± SD OR	1.6 ± 0.5	0.5 ± 0.1	0.5 ± 0.2	2.2 ± 0.9
	Median RD	1.5	0.5	0.5	2.6
	Median OR	1.5	0.5	0.5	2.5
	n RD / *n* OR	35/ 33	35 / 32	35 / 34	21 / 17
CHI	Mean RD ± SD RD	1.9 ± 1.1	0.5 ± 0.3	0.5 ± 0.3	2.09 ± 1.1
	Mean OR ± SD OR	1.7 ± 0.8	0.5 ± 0.2	0.5 ± 0.3	2.09 ± 1.1
	Median RD	1.6	0.5	0.5	2.4
	Median OR	1.5	0.5	0.5	2.4
	n RD / *n* OR	35 / 34	35/ 34	35 / 34	5 /5
GMO	Mean RD ± SD RD	3.7 ± 1.1	1.1 ± 0.3	0.9 ± 0.3	2.09 ± 0.9
	Mean OR ± SD OR	3.6 ± 1.0	1.1 ± 0.3	0.9 ± 0.3	2.10 ± 0.9
	Median RD	3.9	1.0	0.9	1.8
	Median OR	3.9	1.0	0.9	1.8
	n RD / *n* OR	35 / 34	35 / 35	35 / 33	23 / 23
GTP	Mean RD ± SD RD	4.3 ± 1.8	1.1 ± 0.5	1.1 ± 0.5	4.2 ± 2.8
	Mean OR ± SD OR	4.3 ± 1.8	1.2 ± 0.4	1.1 ± 0.5	4.2 ± 2.8
	Median RD	4.3	1.3	1.14	3.4
	Median OR	4.3	1.3	1.1	3.4
	n RD / *n* OR	35 / 35	35 / 34	35 / 35	24 / 24

(B) Ratios

Sampling formulation	Statistical parameter	Ratio			
		Tyr/Trp	Phe/Trp	Phe/Tyr	Trp/Kyn x 10³
AGR	Mean RD ± SD RD	3.3 ± 1.0	1.0 ± 0.4	0.31 ± 0.06	0.3 ± 0.2
	Mean OR ± SD OR	3.1 ± 0.7	1.0 ± 0.3	0.31 ± 0.06	0.3 ± 0.2
	Median RD	3.1	0.9	0.30	0.3
	Median OR	3.0	0.9	0.30	0.3
	n RD / *n* OR	35/ 33	35/ 33	35 / 35	21 /21
CHI	Mean RD ± SD RD	3.5 ± 0.8	1.0 ± 0.4	0.30 ± 0.07	0.5 ± 0.2
	Mean OR ± SD OR	3.5 ± 0.6	1.0 ± 0.2	0.29 ± 0.06	0.5 ± 0.2
	Median RD	3.4	0.9	0.27	0.5
	Median OR	3.4	0.9	0.27	0.5
	n RD / *n* OR	35 / 33	35 / 33	35 / 34	5 / 5
GMO	Mean RD ± SD RD	4.2 ± 1.0	1.2 ± 0.3	0.29 ± 0.04	0.6 ± 0.3
	Mean OR ± SD OR	4.1 ± 0.8	1.2 ± 0.3	0.29 ± 0.04	0.6 ± 0.3
	Median RD	4.1	1.1	0.30	0.4
	Median OR	4.1	1.1	0.30	0.4
	n RD / *n* OR	35 / 34	35 / 34	35 / 35	23 / 23
GTP	Mean RD ± SD RD	4.2 ± 1.0	1.4 ± 0.4	0.28 ± 0.05	0.4 ± 0.3
	Mean OR ± SD OD	4.1 ± 0.9	1.1 ± 0.3	0.28 ± 0.04	0.4 ± 0.3
	Median RD	3.9	1.1	0.27	0.3
	Median OR	4	1.0	0.27	0.3
	n RD / *n* OR	35 / 34	35 / 33	35 / 35	24 / 23

Table S7. Compilation of *p*-values from statistical analyses (one-way ANOVA with multiple testing, Tukey test) of the data on collected analyte amounts from different sampling formulations presented in Figure 3 in the main article. The significance levels used were: *p < 0.05, **p < 0.01, ***p < 0.001.

Sampling Formulation	***p*-value**							
	Tyr	Phe	Trp	Kyn	Tyr/Trp	Phe/Trp	Phe/Tyr	Trp/Kyn
AGR - CHI	0.984 (n.s.)	0.998 (n.s.)	0.981 (n.s.)	0.999 (n.s.)	0.168 (n.s.)	1.000 (n.s.)	0.395 (n.s.)	0.481 (n.s.)
AGR - GMO	<0.001 (***)	<0.001 (***)	<0.001 (***)	0.996 (n.s.)	<0.001 (***)	0.01 (**)	0.408 (n.s.)	0.006 (**)
AGR - GTP	<0.001 (***)	<0.001 (***)	<0.001 (***)	0.0063 (**)	<0.001 (***)	0.353 (n.s.)	0.018 (*)	0.695 (n.s.)
CHI - GMO	<0.001 (***)	<0.001 (***)	<0.001 (***)	1.000 (n.s.)	0.02 (*)	0.013 (*)	1.000 (n.s)	0.919 (n.s)
CHI - GTP	<0.001 (***)	<0.001 (***)	<0.001 (***)	0.098 (n.s.)	0.012 (*)	0.408 (n.s.)	0.524 (n.s.)	0.862 (n.s.)
GMO - GTP	0.084 (n.s.)	0.414 (n.s.)	0.091 (n.s.)	0.001 (**)	1.000 (n.s.)	0.429 (n.s.)	0.497 (n.s.)	0.093 (n.s.)

Table S8. Compilation of analyte amounts collected by different sampling formulations. The quantity of the analyte collected by a particular formulation was divided by the corresponding quantity collected by another formulation from each individual test subjects. The average values from these calculations are shown with standard deviations.

Analyte	GMO/AGR	GMO/CHI	GTP/AGR	GTP/CHI	AGR/CHI	GTP/GMO
Tyr	2.4 ± 0.8 (n = 58)	2.4 ± 0.9 (n = 58)	2.8 ± 1.5 (n = 65)	2.8 ± 1.6 (n = 65)	1.1 ± 0.4 (n = 63)	1.1 ± 0.5 (n = 61)
Phe	2.3 ± 0.9 (n = 64)	2.4 ± 0.8 (n = 64)	2.4 ± 1.3 (n = 66)	2.5 ± 1.3 (n = 66)	1.1 ± 0.4 (n = 64)	1.1 ± 0.6 (n = 68)
Trp	1.8 ± 0.8 (n = 66)	2.0 ± 0.8 (n = 63)	2.1 ± 1.2 (n = 70)	2.3 ± 1.3 (n = 67)	1.2 ± 0.5 (n = 67)	1.3 ± 0.6 (n = 66)
Kyn	1.1 ± 0.7 (n = 20)	1.0 ± 0.7 (n = 6)	1.5 ± 1.0 (n = 13)	1.2 ± 0.4 (n = 6)	1.4 ± 0.6 (n = 6)	2.0 ± 0.9 (n = 18)

Table S9. Compilation of coefficients of variation (CV) of analyte amounts and analyte ratios collected by different sampling formulations. Values shown in the table calculated for the raw data (RD) and data after the statistical outlier removal (OR). CV values were calculated based on the mean values and standard deviations reported in **Table S3**.

Sampling formulation		CV (%)							
		Tyr	Phe	Trp	Kyn	Tyr/Trp	Phe/Trp	Phe/Tyr	Trp/Kyn
AGR	RD	46	40	47	191	30	40	19	93
	OR	35	33	47	55	25	33	19	74
CHI	RD	61	51	59	60	26	36	27	64
	OR	44	41	51	60	20	30	21	64
GMO	RD	32	29	35	48	27	25	14	54
	OR	23	29	32	40	23	25	14	51
GTP	RD	44	42	45	72	26	36	18	83
	OR	44	42	45	70	24	27	18	77

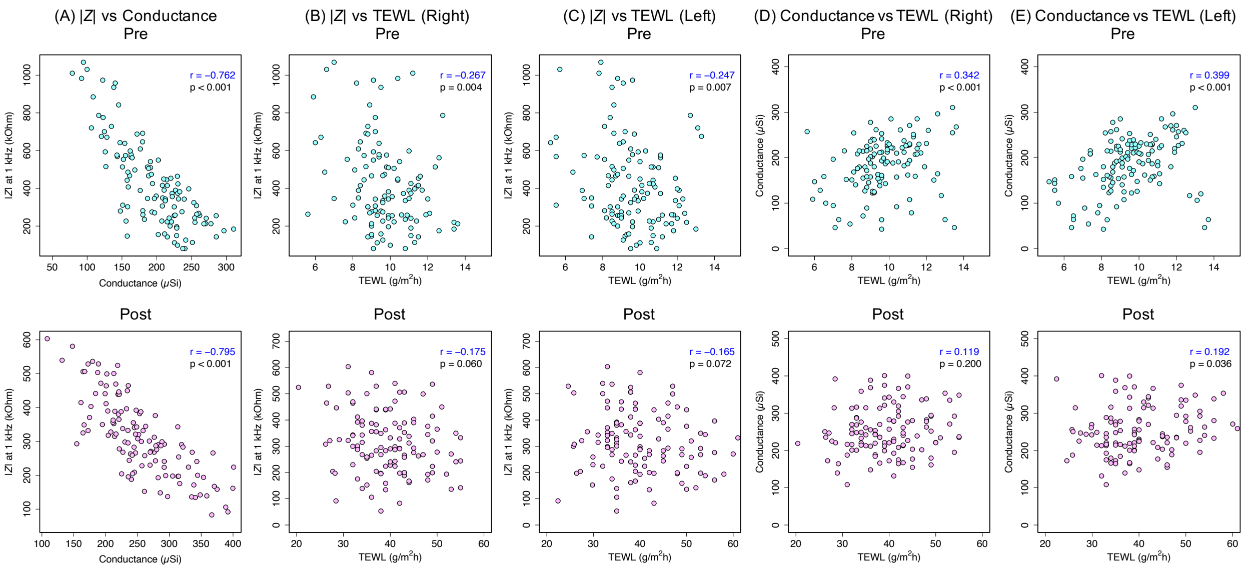

Figure S9. Spearman's correlation plots between biophysical skin parameters measured pre (top panel, cyan symbols) and post (bottom panel, pink symbols) sampling. The correlation between skin resistance (i.e., $|Z|_{1kHz}$) and conductance (i.e., $1/|Z|_{315kHz}$) is shown in (A), the correlation between skin resistance and TEWL assessed on the right arm is shown in (B), while the corresponding correlation for left arms is shown in (C), the correlation between conductance and TEWL measured on right arm is shown in (D), while the corresponding correlation for left arms is shown in (E).

(A) Correlations based on data from all sampling formulations combined

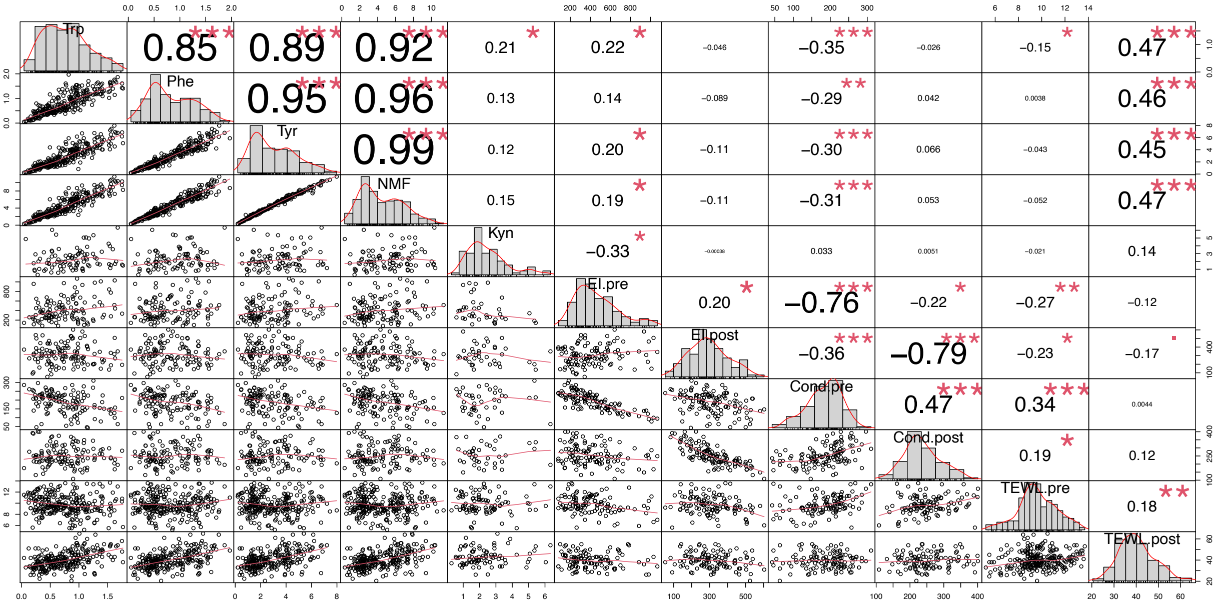

(B) Correlations based on data from the sampling formulation AGR

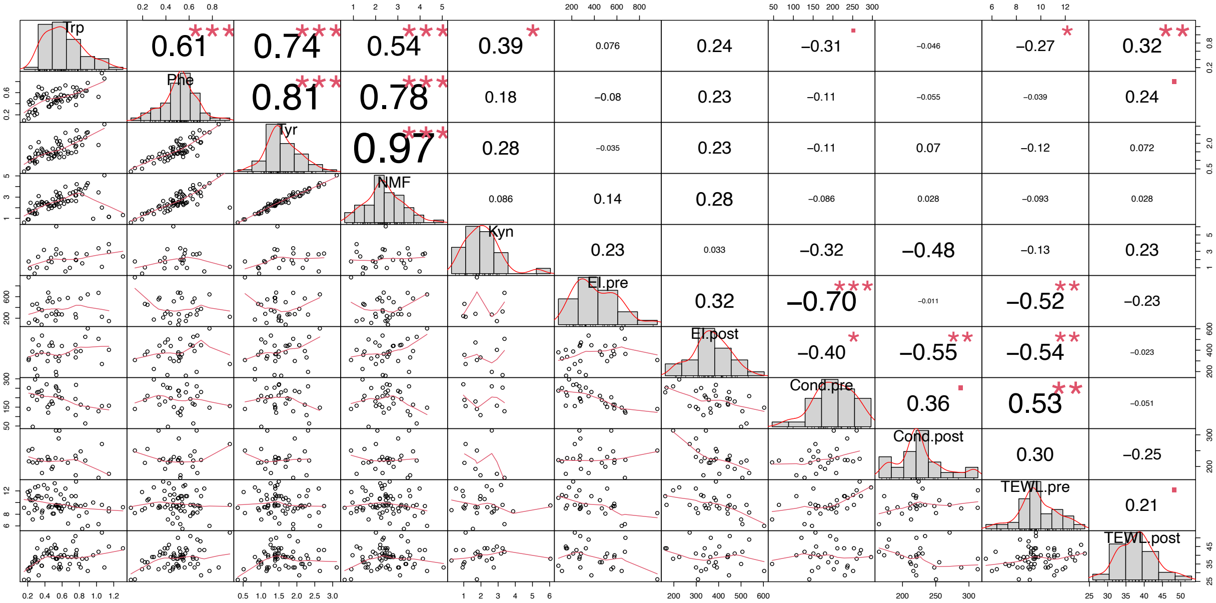

(C) Correlations based on data from the sampling formulation CHI

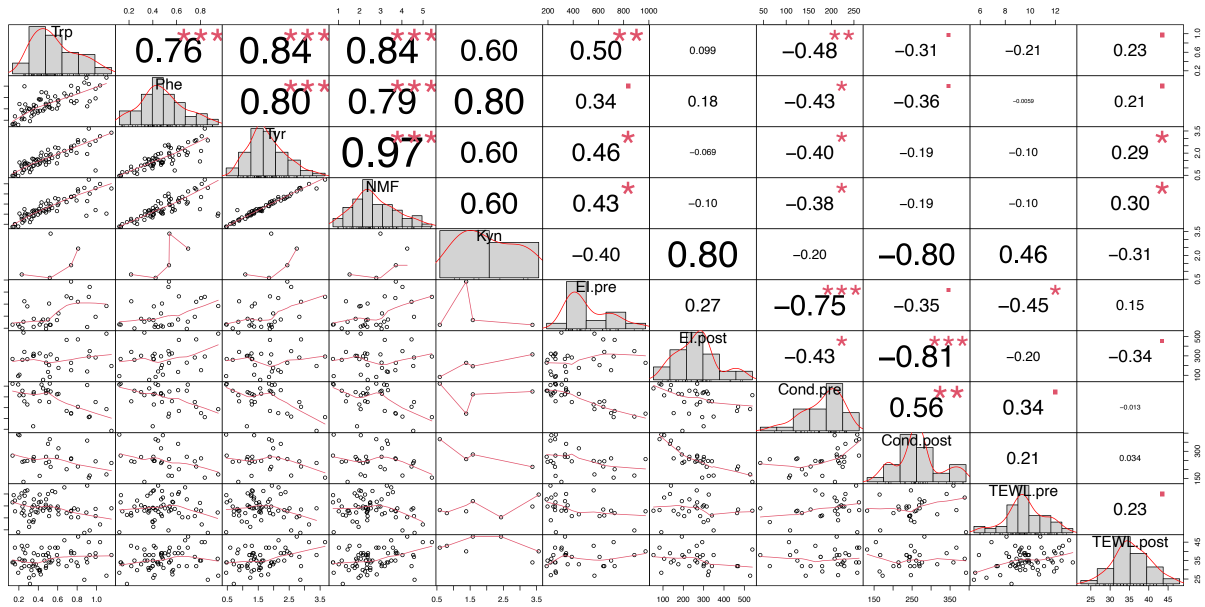

(D) Correlations based on data from the sampling formulation GMO

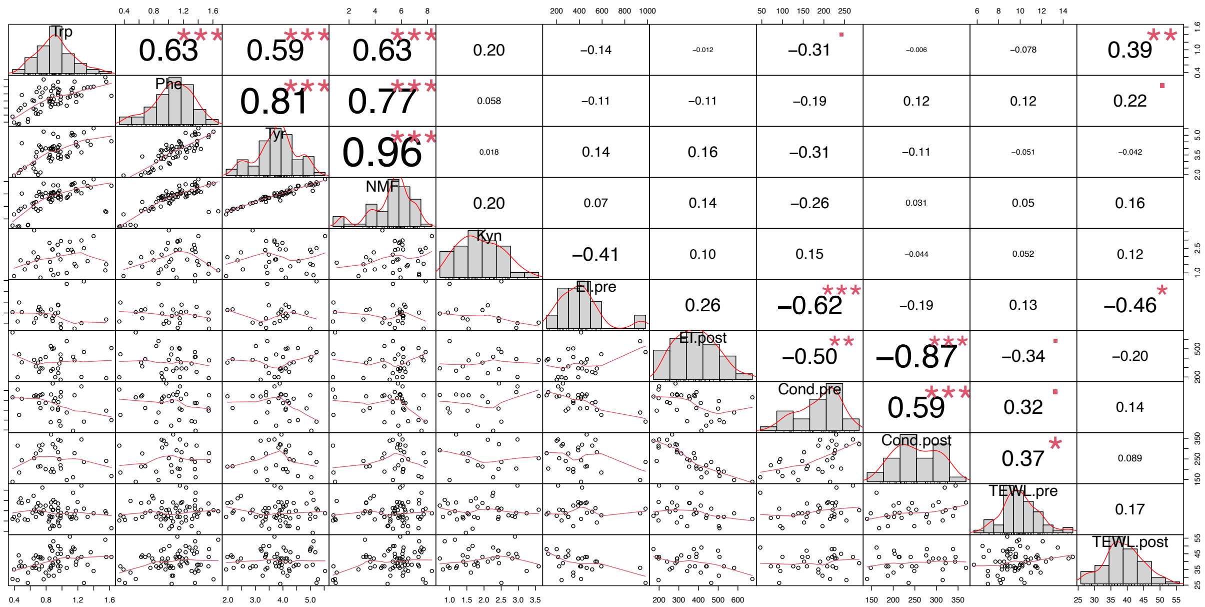

(E) Correlations based on data from the sampling formulation GTP

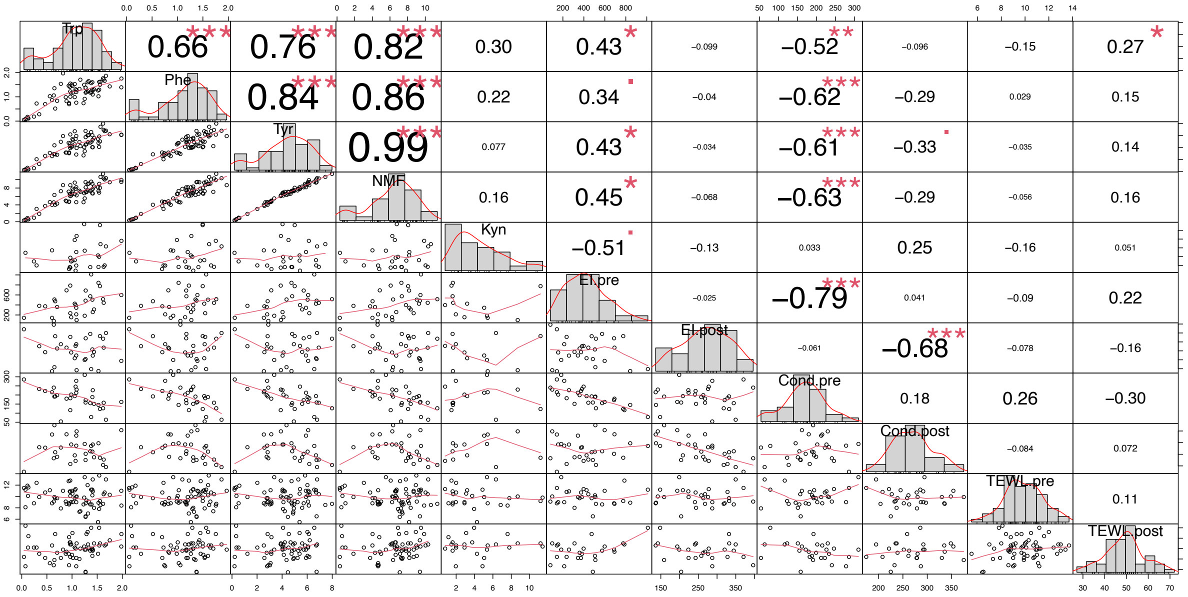

(F) Correlations based on data from the sampling formulations CHI and GTP combined (charged formulations)

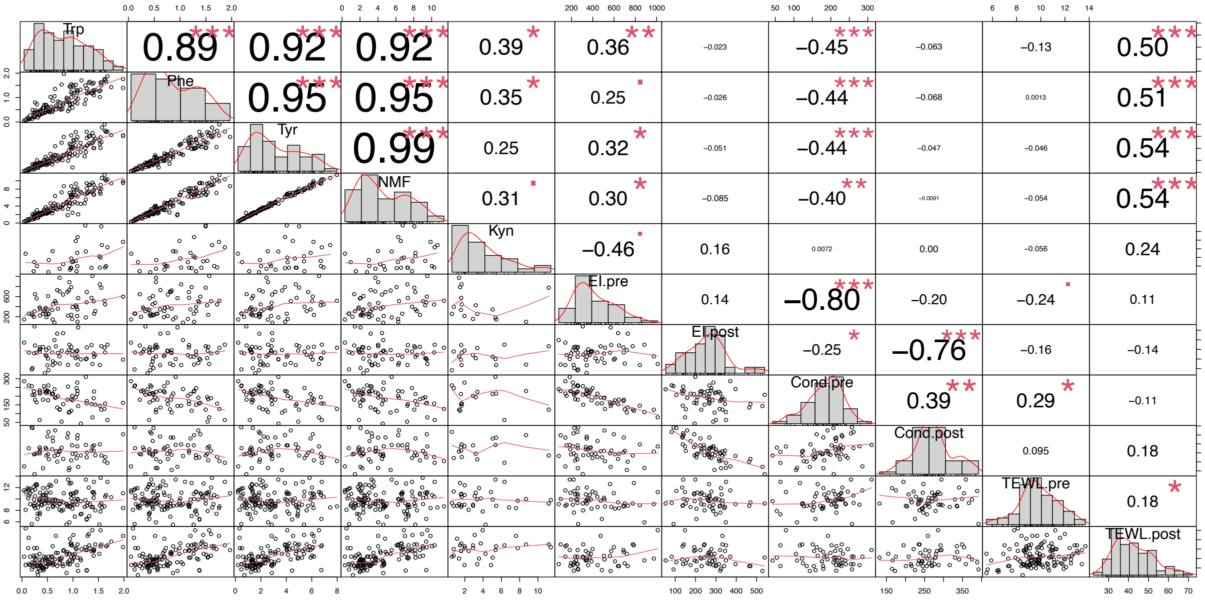

(G) Correlations based on data from the sampling formulations AGR and GMO combined (non-charged formulations)

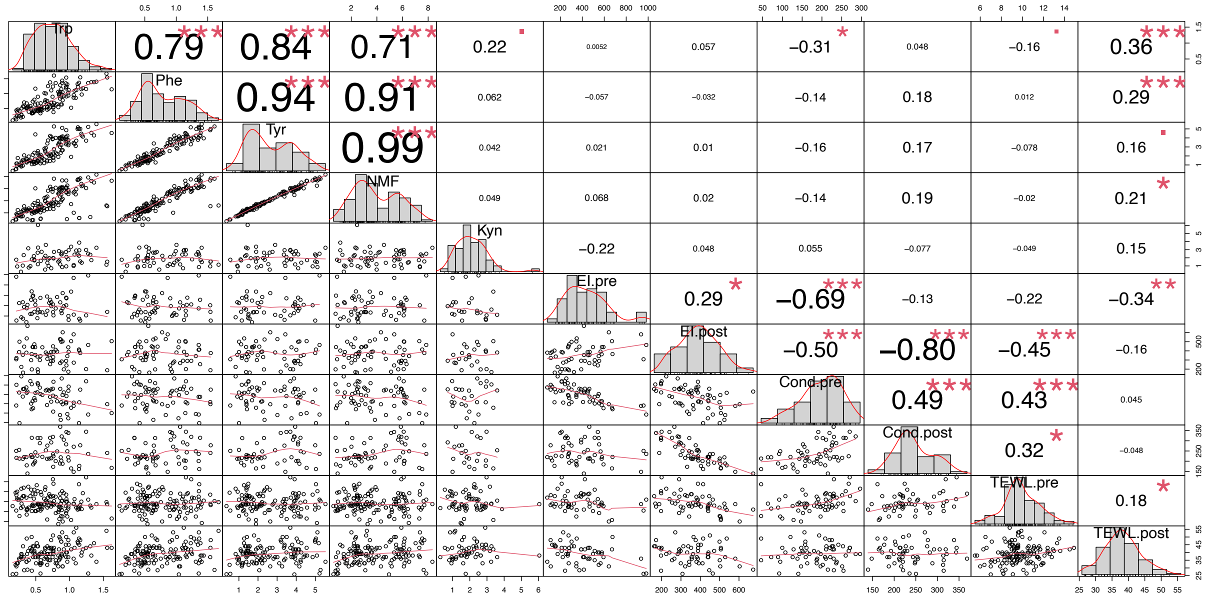

Figure S10. Spearman's correlation plots between the collected amounts of the analytes Tyr, Phe, Trp, and Kyn, including the NMF (i.e., the sum of Trp, Phe and Tyr), and different biophysical skin parameters used to evaluate skin barrier properties (i.e., skin resistance (EI), conductance (Cond.), and transepidermal water loss (TEWL). The skin barrier measurements were performed before (i.e., pre) and after sampling (i.e., post). The quantity of Tyr, Phe and Trp is presented in nmol/cm^2, and the quantity of Kyn is shown in nmol/cm^2 x 10^{-3}. TEWL was measured in g/m^{2}h, EI in kOhm and conductance in μSi. The Spearman's correlation coefficient (r) values are presented as numbers and the significance levels used were: *$p < 0.05$, **$p < 0.01$, ***$p < 0.001$. The correlations were done with RStudio (RStudio v 1.3.1093, PBC, Boston, MA, USA) by using the Performance Analytics package.
